# A genome-wide screening and SNPs-to-genes approach to identify novel genetic risk factors associated with frontotemporal dementia

**DOI:** 10.1016/j.neurobiolaging.2015.06.005

**Published:** 2015-10

**Authors:** Raffaele Ferrari, Mario Grassi, Erika Salvi, Barbara Borroni, Fernando Palluzzi, Daniele Pepe, Francesca D'Avila, Alessandro Padovani, Silvana Archetti, Innocenzo Rainero, Elisa Rubino, Lorenzo Pinessi, Luisa Benussi, Giuliano Binetti, Roberta Ghidoni, Daniela Galimberti, Elio Scarpini, Maria Serpente, Giacomina Rossi, Giorgio Giaccone, Fabrizio Tagliavini, Benedetta Nacmias, Irene Piaceri, Silvia Bagnoli, Amalia C. Bruni, Raffaele G. Maletta, Livia Bernardi, Alfredo Postiglione, Graziella Milan, Massimo Franceschi, Annibale A. Puca, Valeria Novelli, Cristina Barlassina, Nicola Glorioso, Paolo Manunta, Andrew Singleton, Daniele Cusi, John Hardy, Parastoo Momeni

**Affiliations:** a Department of Molecular Neuroscience, Institute of Neurology, UCL, London, UK; b Laboratory of Neurogenetics, Department of Internal Medicine, Texas Tech University Health Science Center, Lubbock, TX, USA; c Department of Brain and Behavioural Sciences, Medical and Genomic Statistics Unit, University of Pavia, Pavia, Italy; d Department of Health Sciences, University of Milan at San Paolo Hospital, Milan, Italy; e Neurology Clinic, University of Brescia, Brescia, Italy; f Neurology I, Department of Neuroscience, University of Torino and Città della Salute e della Scienza di Torino, Turin, Italy; g Molecular Markers Laboratory, IRCCS Istituto Centro San Giovanni di Dio Fatebenefratelli, Brescia, Italy; h Neurology Unit, Department of Pathophysiology and Transplantation, University of Milan, Fondazione Cà Granda, IRCCS Ospedale Policlinico, Milan, Italy; i Division of Neurology V and Neuropathology, Fondazione IRCCS Istituto Neurologico Carlo Besta, Milano Italy; j Department of Neuroscience, Psychology, Drug Research and Child Health, University of Florence, Florence, Italy; k Neurogenetic Regional Centre ASPCZ Lamezia Terme, Lamezia TErme, Italy; l Department of Clinical Medicine and Surgery, University of Naples Federico II, Naples, Italy; m Geriatric Center Frullone—ASL Napoli 1 Centro, Naples, Italy; n Department of Neurology, IRCCS Multimedica, Milan, Italy; o Department of Medicine and Surgery, University of Salerno, Baronissi, Salerno, Italy; p Cardiovascular Research Unit, IRCCS Multimedica, Milan, Italy; q Department of Molecular Cardiology, IRCCS Fondazione S. Maugeri, Pavia, Italy; r Hypertension and Related Disease Centre, AOU-University of Sassari, Sassari, Italy; s Chair of Nephrology, Nephrology and Dialysis and Hypertension Unit, San Raffaele Scientific Institute, Università Vita Salute San Raffaele, Milano, Italy; t Laboratory of Neurogenetics, National Institute on Aging, National Institutes of Health, Bethesda, MD, USA; u Institute of Biomedical Technologies, Italian National Research Council, Milan, Italy

**Keywords:** Frontotemporal dementia, Association study, Case-control, Genetic risk factors, Functional annotation, Population

## Abstract

Frontotemporal dementia (FTD) is the second most prevalent form of early onset dementia after Alzheimer's disease (AD). We performed a case-control association study in an Italian FTD cohort (n = 530) followed by the novel single nucleotide polymorphisms (SNPs)-to-genes approach and functional annotation analysis. We identified 2 novel potential loci for FTD. Suggestive SNPs reached *p*-values ∼10^−7^ and odds ratio > 2.5 (2p16.3) and 1.5 (17q25.3). Suggestive alleles at 17q25.3 identified a disease-associated haplotype causing decreased expression of –cis genes such as *RFNG* and *AATK* involved in neuronal genesis and differentiation and axon outgrowth, respectively. We replicated this locus through the SNPs-to-genes approach. Our functional annotation analysis indicated significant enrichment for functions of the brain (neuronal genesis, differentiation, and maturation), the synapse (neurotransmission and synapse plasticity), and elements of the immune system, the latter supporting our recent international FTD–genome-wide association study. This is the largest genome-wide study in Italian FTD to date. Although our results are not conclusive, we set the basis for future replication studies and identification of susceptible molecular mechanisms involved in FTD pathogenesis.

## 1 Introduction

Frontotemporal dementia (FTD) is the second most prevalent form of early onset dementia after Alzheimer's disease (AD), worldwide (Ratnavalli et al., 2002). The 2 main FTD syndromes affect an individual's behavior or language resulting either in the behavioral variant FTD (bvFTD) or the language variant broadly named primary progressive aphasia (PPA) (Gorno-Tempini et al., 2011, Rascovsky et al., 2011). The latter is subdivided into semantic variant PPA and non-fluent/agrammatic variant PPA (Gorno-Tempini et al., 2011). Each of these syndromes is distinct and is characterized by specific signatures (Ferrari et al., 2013). FTD can also overlap with motor neuron disease (MND) resulting in the broad subgroup called FTD-MND (Strong et al., 2009).

The genetics of FTD have become clearer over the past 15 years when mutations in the microtubule associated protein tau (*MAPT*) and progranulin (*GRN*) genes were identified (Baker et al., 2006, Cruts et al., 2006, Hutton et al., 1998); more recently, a repeat expansion in the *C9orf72* gene has been reported in the FTD–amyotrophic lateral sclerosis spectrum (DeJesus-Hernandez et al., 2011, Renton et al., 2011) and a small number of FTD cases (<5% all together) has been associated with variability in a handful of genes including the transactive response DNA binding protein 43 (TDP-43) and valosin containing protein (Ferrari et al., 2014, Rohrer and Warren, 2011). Recently, international genome-wide association studies (GWAS) identified novel potential risk factors for FTD with TDP-43 pathology such as the transmembrane protein 106B (*TMEM106B*) gene (Van Deerlin et al., 2010), and the locus containing the RAB38, member RAS oncogene family (*RAB38*) and catepsin C (*CTSC*) genes for bvFTD, and the *HLA* locus for the FTD spectrum (Ferrari et al., 2014).

Currently, there are no comprehensive epidemiological data on monogenic FTD in the Italian population. However, the majority of FTD cases has been associated with *GRN* mutations (Benussi et al., 2009, Borroni et al., 2010), whilst only a few cases with *MAPT* (Alberici et al., 2004, Binetti et al., 2003). In addition, a few cases have been associated with mutations in *TDP-43* (Borroni et al., 2010) and no proper epidemiological data yet exist on *C9orf72* variants (Benussi et al., 2014, Galimberti et al., 2014, Ticozzi et al., 2014). For the vast majority of cases in Italy, the common genetic underpinnings of the disease are still unknown.

As we had access to genome-wide genotyping data for > 600 Italian FTD cases, we intended to better characterize the genetic underpinnings of FTD in this population. Here, we present the results of our analysis of genome-wide markers in the classical association and the novel SNPs-to-genes fashions. In addition, we also performed functional annotation of the suggestive genes that we identified.

## 2 Materials and methods

### 2.1 Samples

#### 2.1.1 Cases

Genotyping data of DNA samples diagnosed with FTD were available to us from the FTD-GWAS data set (Ferrari et al., 2014); specifically, we had access to raw data of 634 samples, which were obtained from 8 Italian research centers (Supplementary Table 8). After quality check (QC) steps 530 patients diagnosed with bvFTD (n = 418), semantic variant PPA (n = 27), agrammatic variant PPA (n = 61), and FTD-MND (n = 23) were included in the study. Mean (± standard deviation [SD]) age of onset was 64.1 ± 20.7 years (range, 29.0–87.0) with male-to-female ratio 243/287. Four hundred eighty-two of 530 cases had been characterized for candidate genes: a minority of cases carried variants in *MAPT* (n = 2; 0.4%), *GRN* (n = 37; 7.7%), and *C9orf72* (n = 27; 5.6%). Three cases (2 bvFTD and 1 FTD-MND) had double variants (*C9orf72* and *GRN*). The cases with variants in *MAPT* and *GRN* were kept in the study because these were nonpathogenic polymorphisms. Conversely, all cases with known pathogenic mutations in *MAPT* and *GRN* were excluded from the study a priori, whereas those carrying *C9orf72* expansions were kept because we adopted here the same strategy as in the international FTD-GWAS (Ferrari et al., 2014). All cases were diagnosed according to the Neary criteria (Neary et al., 1998) and/or the more recent Rascovsky and Gorno-Tempini criteria (Gorno-Tempini et al., 2011, Rascovsky et al., 2011). The cases were collected and genotyped at the University College London by means of Illumina human 660K-Quad Beadchips assayed on the Illumina Infinium platform (Illumina, San Diego, CA, USA).

#### 2.1.2 Controls

The control sample used in the present study has been collected during the HYPERGENES project (European Network for Genetic-Epidemiological Studies; www.hypergenes.eu) (Salvi et al., 2012). The sample set (n = 1327; 926 after QC) included 349 (37.7%) women and the mean (±SD) age was 58.2 ± 6.1 years (range, 50.0–97.0). All participants were unrelated, collected in Italy, and of Caucasian ancestry. All subjects had no abnormal findings on physical and neurological examination. The control samples were genotyped at the University of Milan, using the Illumina 1M-duo array.

Written informed consent from patients and control individuals was obtained at every site by the principal investigator. Each study site obtained approval from a local ethics committee (UK ethics committee number 10/H0716/3, ethics committee of the University of Milan approval 24/04/2008) or institutional research board; every participating group provided consent for the use of the samples to pursue the goals of this study.

### 2.2 Association and expression quantitative trait loci analyses

All QC steps were performed in accordance with the protocol written by C.A Anderson (Anderson et al., 2010).

We assessed population structure using principal components analysis (PCA) as implemented in the Golden Helix software (http://www.goldenhelix.com/) to infer continuous axes of genetic variation. We ruled out relatedness across subjects (cases and controls) through identity-by-descent analysis, as implemented in PLINK, for all possible pairs of individuals. After these QC steps, we found that none of the cases and controls were related. We excluded outlier samples defined as individuals exceeding a default number of SDs (6.0) from the whole sample. Then, we used markers of highest quality to impute approximately 2.5 million SNPs, based on European-ancestry haplotype reference (HapMap II CEU population build 36, release 22). Imputed SNPs with a minor allele frequency (MAF) < 0.01 and with low imputation quality (Rsq < 0.80) were removed. We then computed each SNP *p* value with a logistic regression of FTD status on SNP dosage levels, adjusting for sex, and the first 4 principal components. Imputation and single-marker significance were performed using mach2dat software (Li et al., 2010).

Three polymorphisms (rs17650901, rs1052553, and rs17652121) throughout the *MAPT* gene are in complete linkage disequilibrium with each other and are inherited as 2 separate haplotypes, H1 and H2. For each sample, we constructed the *MAPT* haplotypes and we performed case and/or control haplotype associations to test for association with disease. The logistic regressions are corrected for sex and the first 4 principal components.

Suggestive SNPs were investigated for potential effects on expression quantitative trait loci in brain using the online freely available database braineac (http://www.braineac.org/; accessed in December 2014). This data set allows identification of effects in cis only (±1 Mb from each SNP of interest). The significance threshold is *p*-value ≤ 10^−5^, as recommended by the database curators.

We also used in the GenomeStudio data analysis software (Illumina) to perform loss of heterozygosity and copy number variant (CNV) analysis for the suggestive loci: we evaluated the LogR ratio and B Allele Frequency and visualized CNVs within the chromosomal browser heat map in GenomeStudio.

### 2.3 SNPs-to-genes analysis

To map the SNPs-to-genes, containing genomic coordinates for all genes according to positions on the Genome Browser, hg18 (NCBI assembly GRCh36) were downloaded from the PLINK ftp server [http://pngu.mgh.harvard.edu/∼purcell/plink/res.shtml#hapmap] (accessed in September 2014). SNPs were assigned to a gene if they located within its primary transcript (intragenic region) or 5 kilobases (kb) upstream or downstream of the gene start or end. This was done based on the following reasons: (1) small windows prevent the assignment of an SNP to multiple genes because of reduced overlap of flanking regions; (2) a small percentage of SNPs beyond 10 kb are in moderate to high linkage disequilibrium (LD) with the SNPs within a gene (Petersen et al., 2013); (3) adding SNPs mapped farther away from a gene (20 kb) will include noncausal SNPs and increment noise signals that will eventually impact the power of gene-based tests (Petersen et al., 2013). In total, 965,052 SNPs (42% of the 2,292,247 SNPs of the SNPs imputed during association analysis) were assigned to 18,011 protein coding genes (95.5% of the 18,867 of the PLINK list).

We used an innovative approach to investigate these data by means of 3 different gene-based statistical methods: (1) the extended Simes' procedure (GATES) with SNP pruning based on MAF >0.01; (2) the supervised PCA (sPCA) test with SNP pruning based on MAF >0.01 and SNP log-likelihood > 75%, and; (3) the sequential kernel machine association test (SKAT) without SNP pruning. The *p* values of our genome-wide classical association analysis as well as raw genotyping data were used to perform these analyses.

The GATES method (Li et al., 2011) is an extension of the Simes' approach (Simes, 1986) that combines multiple single marker genotype-phenotype tests (*p* values) applied to each SNP. Briefly, a gene-representative *p* value is derived from the SNP *p* values of the primary association analysis using effective numbers of independent SNP *p* values. In this fashion the gene *p* values are combined to control for the SNP correlation structure that is estimated by the pairwise LD between the SNPs computed on the current cohort under study. An advantage of GATES is that if only SNP-based *p* values are accessible, LD information from a known reference population (e.g., HapMap) can be used to account for the absence of individual genotype information. Thus, GATES is a meta-analysis (post GWAS) method that does not require raw individual phenotype and genotyping data.

The sPCA method (Bair et al., 2006) combines the SNPs that fall within the gene set, based on the genotype calls; then instead of performing PCA (Ma and Dai, 2011) on all SNPs within the gene, estimated PCs are calculated from a subset of SNPs. Because the subset of SNPs is selected using outcome (case-control status) information, it is a supervised procedure. The assumption behind the sPCA is that given a priori defined group of SNPs, only a subset of these SNPs is associated with a latent variable, which then varies with outcome. sPCA uses the first PC score (PC1), also called “eigengene”, to estimate the latent variable. Subsequently, the *p* value of the PC1 effect in a logistic regression evaluates the association between the eigengene and disease outcome. We used the likelihood percentile filter with a threshold of 75% as an SNP pruning method (Spencer et al., 2014). This ranks the log-likelihood values, from a univariate logistic model with case-control status as outcome and SNP status as predictor and filters a prespecified number or proportion of SNPs, that is, our threshold of 75% retains the top ranked 25% of SNPs.

The SKAT approach (Wu et al., 2010) is a regression method to test for association between genetic variants (common and rare) in a region. It considers a logistic kernel-machine model (Liu et al., 2008) for the joint effect of the SNPs in the gene set. The SNPs influence outcome through a general nonlinear kernel function K(S, S), which is an arbitrary function that measures the genomic similarity between the genotypes of the SNPs in the gene set and disease risk. Some commonly used kernels include linear, identity-by-descent and quadratic kernels. Many genotyped SNPs and rare variants (MAF < 0.01) are correlated, and thus the logistic kernel-machine test has good power to detect a significant gene-set effect without an initial pruning. Treating K(S, S) as subject-specific random effects, SKAT uses a score-based variance-component test (with only 1 degree of freedom) to evaluate association between the SNPs in the gene set and the case-control status. We set K(S, S) as the linear kernel to calculate *p* values analytically, thus SKAT can easily be applied to analyse genome-wide data.

Once nominal gene *p* values are obtained from GATES, sPCA and SKAT, we next calculate adjusted *p* values to control for false discovery rate (FDR) using Benjamini and Hochberg (Benjamini and Hochberg, 1995) correction procedure. The significance cutoff level of *p* < 0.05 was used.

To implement the pipeline analysis, we developed our own custom codes of the R v3.1.2 software (www.r-project.org/) by using the R packages SNPRelate, postgwas, spc, assotestR, multtest for read PLINK GWAS files, compute LD, perform GATES, sPCA, and SKAT analysis, and list adjusted *p* values, respectively.

### 2.4 Gene ontology analysis

To assess the biological relevance of the suggestive genes identified through the association and the SNPs-to-genes analyses, functional annotation analysis of gene ontology (GO) terms (Ashburner et al., 2000) was performed.

First, a list of disease-associated genes (DAGs) (see Supplementary Table 4; Supplementary Fig. 4g), including suggestive genes as per association analysis with *p*-value < 10^−4^and genes derived from the GATES + sPCA + SKAT analyses with *p*-value < 0.1 (after FDR correction), was selected and processed by means of DAVID (the Database for Annotation, Visualization, and Integrated Discovery) (Huang da et al., 2009) for functional annotation clustering, considering all GO categories (molecular function [MF], biological process [BP], and cellular component [CC]). DAVID used an agreement score (kappa statistics) matrix and a fuzzy heuristic algorithm (Huang et al., 2007) to cluster functionally similar GO terms associated with DAGs into groups. The highest clustering stringency was used, as this generates fewer functional groups with stronger association between the genes included in each group. An “enriched” *p* value was assigned to each term by comparing the selected gene list to the whole human genome by a modified Fisher's exact test. For each cluster an enrichment score (ES) = −log_10_ (geometric mean of the term *p* values in the cluster) was computed, and the clusters with an ES ≥ 1.3 or greater were highlighted, as this is equivalent to nonlog scale of *p* value of 10^−1.3^ = 0.05.

Second, accounting for the hierarchical (parent-child) structure of GO, and to reduce redundancy, we performed a gene-set enrichment analysis for each GO category (BP, CC, and MF) using the Kolmogorov-Smirnov test as implemented in Alexa et al. (2006). This ranks all the *p* values of the gene-based analysis and then tests an ES based on the maximum ranking deviation of the genes annotated in a GO term from a random gene set uniformly distributed, as expected by chance. This was done for all GO terms. We used gene *p* value as the minimum *p* value from the GATES, sPCA, or SKAT *p* values. In addition, we selected the option “weight01”, a GO term decorrelation, which considers only the best fitted GO-term, removing redundancy between reported terms. Specifically, weight01 method is a heuristic combination of “elim” and “weight” methods. The former investigates the GO nodes from the bottom to the top of the GO hierarchy to ensure that the most specific nodes are scored, and iteratively removes the genes mapped to significant GO terms before scoring the parent nodes. The latter investigates the GO nodes bottom up such as the elim method but, instead of removing the genes once a significant node has been scored, it assigns weights based on the scores of neighboring GO terms to each gene.

As for the gene-based pipeline, GO analysis was performed by our own custom R codes with packages RDAVIDWebService, and topGO for DAVID clustering, and gene set enrichment analysis, respectively.

## 3 Results

### 3.1 Association and expression quantitative trait loci analyses

Our association analysis was performed on 530 FTD cases and 926 controls. We did analyze the entire cohort, whereas we could not analyze each subtype separately because of statistical power issues.

#### 3.1.1 Analysis of novel genetic markers

**Fig. 1:**
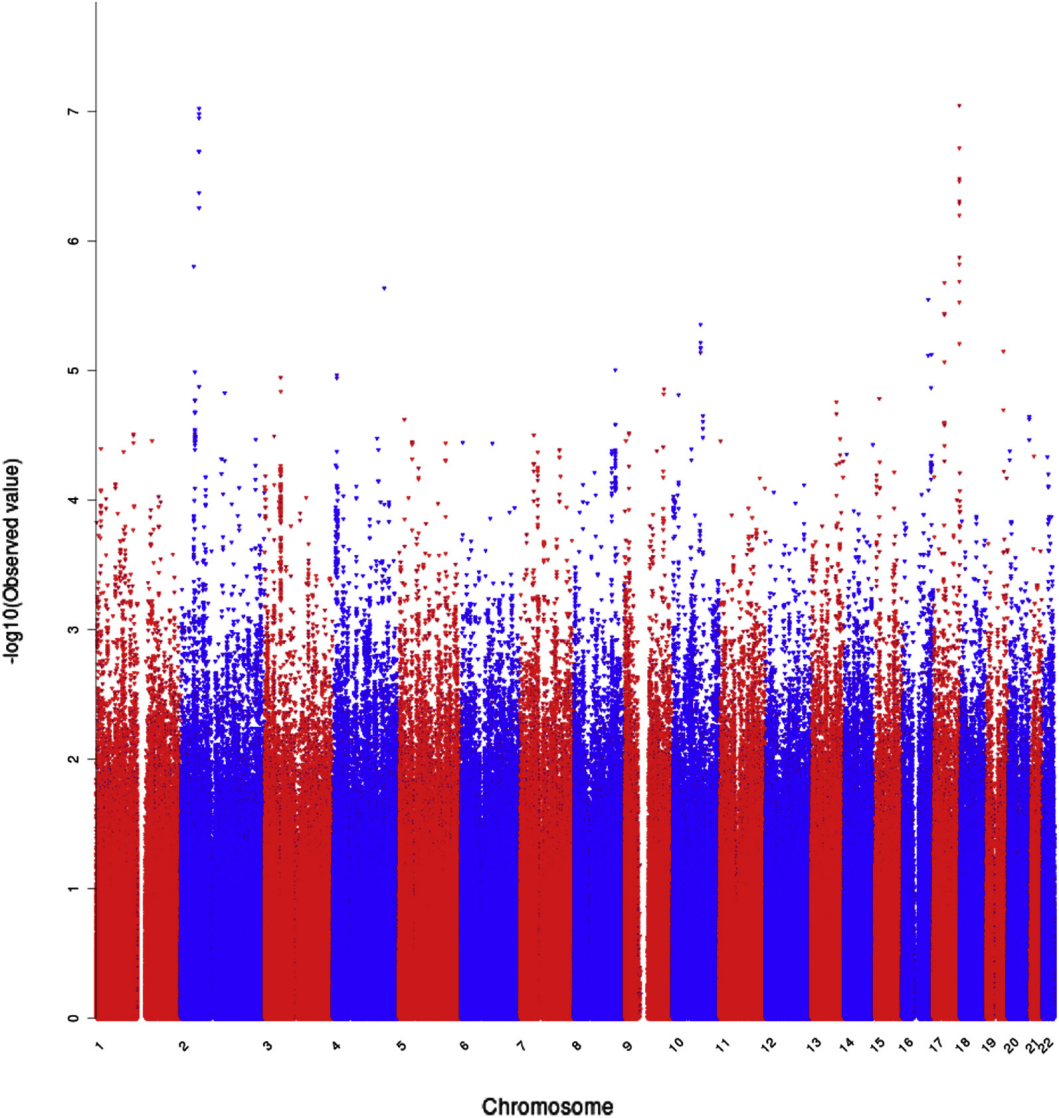
Manhattan plot of the association analysis. The genome-wide significance level threshold was: *p*-value = 2.18 × 10^−8^. Single nucleotide polymorphisms on chromosomes 2 and 17 reached strongly suggestive *p* values. (For interpretation of the references to color in this figure legend, the reader is referred to the Web version of this article.)

We did not identify SNPs that reached the genome-wide significance level (*p*-value < 2.18 × 10^−8^). However, considering as suggestive threshold the *p*-value ≤ 10^−5^ (Supplementary Table 1), as supported by the quantile-quantile plot (Supplementary Fig. 1), a number of SNPs mapping to chromosomes (chr) 2 and 17 were strongly suggestive (Table 1; Fig. 1).

**Fig. 2:**
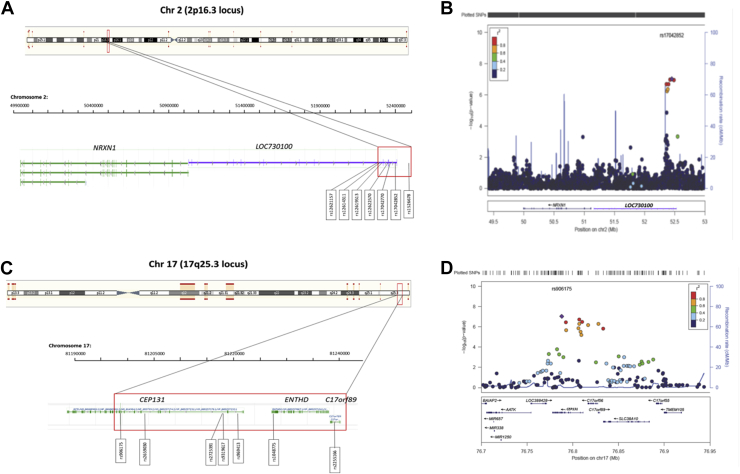
Suggestive loci and regional plot analysis. (A) Highlight of the 2p16.3 locus holding 7 suggestive single nucleotide polymorphisms (SNPs) within and downstream of *LOC730100*. (B) Regional plot for the 2p16.3 locus. The 7 suggestive SNPs are in high linkage disequilibrium (LD) with each other but no other SNPs in the surrounding region. (C) Highlight of the 17q25.3 locus holding 7 suggestive SNPs across *CEP131*, *ENTHD,* and *C17orf89*. (D) Regional plot for the 17q25.3 locus. The 7 suggestive SNPs are in high LD with 5 further SNPs at this locus identifying a 45.2 Kb long haplotype block. In regional plot, each circle represents a SNP, y-axis is the −log10 association *p* value for frontotemporal dementia association and x-axis represents the physical position on the chromosome (build 36, hg18). The circles are filled with colors according to the linkage disequilibrium (LD; r2) between the given SNP and the lead SNP (purple square). (For interpretation of the references to color in this figure legend, the reader is referred to the Web version of this article.)

All suggestive SNPs mapping to the short arm of chr 2 (2p16.3 locus) located to introns of the uncharacterized gene *LOC730100* (Fig. 2A). For each suggestive SNP the minor alleles showed odds ratios (OR) exceeding 2.5 (Table 1). Regional plot analysis at this locus (Fig. 2B) revealed that the SNPs clustering within *LOC730100* are in strong LD with each other but not with any surrounding SNP (0.5 Mb centromeric and 3 Mb telomeric) suggesting high rates of recombination at this locus. We evaluated effects on transcription exerted by the suggestive SNPs at the 2p16.3 locus: data were only available for the risk allele of rs12619513 (*LOC730100*), revealing no significant in cis effects on transcription in any of the assayed brain tissues (Supplementary Table 2). *LOC730100* is immediately located downstream (centromeric) from the neurexin 1 (*NRXN1*) gene (Fig. 2A); based on our data, no SNPs within *NRXN1* resulted significant or suggestive neither they were in LD with the suggestive SNPs found in *LOC730100*. *NRXN1* is involved in neurodevelopment and it was shown being polymorphic, especially, carrying CNVs associated with a spectrum of neurobehavioral and neuropsychiatric disorders (Bena et al., 2013). The visual analysis of the distribution of the SNPs at the *NRXN1* locus through the Illumina Genome Viewer in GS did not provide evidence for CNVs within *NRXN1* in our sample set; of note, when the B allele frequency plot suggested the possibility of a CNV this was not supported by the LogR ratio plot (see 2 examples in Supplementary Fig. 2a and 2b).

The suggestive SNPs mapping to the long arm of chr 17 (17q25.3locus) located to the introns of 2 genes, the centrosomal protein 131 (*CEP131*) and the yet uncharacterized *C17orf89*, and to the 3′-UTR of the ENTH domain containing 2 (*ENTHD2*) gene (Fig. 2C). The OR for the risk alleles barely exceeded 1.5 (Table 1). Regional plot analysis at this locus revealed that 12 SNPs (rs906175, rs2659030, rs9912789, rs9896850, rs2725391, rs9319617, rs12939525, rs8073077, rs969413, rs1048775, rs2255166, and rs2659005) spanning a genetic region of ∼45.2 Kb comprising the *CEP131*, *ENTHD2*, and *C17orf89* genes are in moderate to strong LD (Fig. 2D; Table 2), suggesting that a haplotype substructure at this locus might associate with the disease in this population. Our haplotype analysis showed that either in the case of the 12 SNPs in LD (rs906175, rs2659030, rs9912789, rs996850, rs2725391, rs9319617, rs12939525, rs8073077, rs969413, rs1048775, rs2255166, and rs2659005) or the 7 SNPs highlighted by our association analysis (rs906175, rs2725391, rs969413, rs2659030, rs2255166, rs9319617, and rs1048775) there was: (1) a risk haplotype substructure suggestive of association with disease: TACCTTTCACCT with *p*-value = 1.60 × 10^−5^ and OR = 1.42, and TATACTC with *p*-value = 4.39 × 10^−6^ and OR = 1.45, respectively (Table 2), and; (2) a protective haplotype substructure suggestive of association with the controls: CGTTCCCTTGTC with *p*-value = 7.42 × 10^−6^ and OR = 0.695, and CGCTGCT with *p*-value = 8.30 × 10^−7^ and OR = 0.67, respectively (Table 2). We then analyzed the potential effects exerted on transcription by the suggestive SNPs (rs906175, rs2725391, rs969413, rs2659030, rs2255166, rs9319617, and rs1048775) at this locus: significant in–cis effect was seen for rs906175 (risk allele T; *p*-value = 5.6 × 10^−6^) and rs2659030 (risk allele A; *p*-value = 7.7 × 10^−6^) both associating with decreased expression of the radical fringe (*RFNG*) gene in the hippocampus (Supplementary Table 3; Supplementary Fig. 3a). Other 4 SNPs (rs2725391, rs969413, rs2255166, and rs9319617) showed that their risk alleles were suggestive for in–cis effects also on decreased expression of *RFNG* in the hippocampus (Supplementary Table 3). Six SNPs (rs906175, rs2725391, rs969413, rs2659030, rs9319617, and rs1048775) showed that their risk alleles had suggestive effects on decreased expression of the apoptosis-associated tyrosine kinase (*AATK*) gene and the microRNA 1250 (*MIR1250*) in the temporal cortex (Supplementary Table 3; Supplementary Fig. 3b). Taken together, these data are of relevance for a number of reasons: (1) they reveal a suggestive risk haplotype on chr 17 encompassing the 3 genes *CEP131*, *ENTHD2*, and *C17orf89* in our cohort, and; (2) each of the suggestive SNPs affects transcription in–cis: particularly, all the risk alleles of the suggestive SNPs cause a regional-specific decrease in expression of the cis genes *RFNG* (hippocampus) and *AATK* and*MIR1250* (temporal cortex) (Supplementary Table 3; Supplementary Fig. 3a and 3b) lending support to the idea that, if on one hand the effect size of each single suggestive SNP is rather small, on the other their cumulative effect on transcription processes might be the biological mechanism underlying the association at this locus.

#### 3.1.2 Stratified analyses

We evaluated whether any SNP with 10^−4^ < *p*-value < 10^−8^ (Supplementary Table 1) differently associated with disease when stratified by sex and the *MAPT* haplotypes.

Our analysis revealed that 2 SNPs, rs3110642 and rs3110643, significantly (*p*-values = 0.022 and 0.027, respectively) associated with FTD in the female population (Table 3). Both SNPs map to chr 17 and locate to the introns of the HNF1 homeobox B (*HNF1B*) gene; of note, their OR is almost twice bigger in females comparatively to men (OR = 2.15 vs. 1.32 and OR = 2.12 vs. 1.33; see Table 3).

The *MAPT* haplotypes were differently distributed in our cases and controls (Table 4). Neither the H1/H1 nor the H1/H2 haplotype combinations significantly associated with disease, whereas H2/H2 was significantly over-represented in the controls (*p*-value = 0.025; OR = 0.54) thus resulting protective (Table 4). We did not observe any influence of any of the *MAPT* haplotypes on the suggestive or nominally significant SNPs, that is, the results observed in the association analysis were independent from the *MAPT* haplotype status.

#### 3.1.3 Analysis of candidate genetic markers

We then verified the relevance of other genetic risk factors that have previously been associated with FTD and/or closely related forms of neurodegenerative disease (Table 5). We first evaluated the SNPs in *TMEM106B* (Van Deerlin et al., 2010) and those identified in the recent international FTD-GWAS for *RAB38*, *CTSC,* and the *HLA* locus (Ferrari et al., 2014); then we assessed the markers defining the *C9orf72* (van Es et al., 2009), *MAPT* (Höglinger et al., 2011), and *TOMM40/APOE* (Seshadri et al., 2010) loci.

It is relevant to note that none of the international GWAS hits (Ferrari et al, 2013, Deerlin et al., 2010) was replicated in the Italian cohort reaching *p* values of ∼3–5 × 10^−1^ and OR ∼1.04 for the risk alleles of *TMEM106B* (Table 5), 6–7 × 10^−1^ and OR ∼1.05 for the *RAB38*/*CTSC* risk alleles (Table 5) and 1–2 × 10^−1^ and OR ∼1.1 for the *HLA* locus risk markers (Table 5). When we assessed the current suggestive SNPs in previous FTD-GWAS these held nonsignificant *p* values (4–7 × 10^−1^; see Supplementary Table 4). These data suggest that risk factors that associate with FTD cases with European ancestry but of Western/Central/North-European and North-American extraction seem not to associate with South-European/Mediterranean population and (apparently) vice versa.

Similar results were obtained for the risk alleles at the *C9orf72* (*p*-value = 3 × 10^−2^, OR = 1.2) and *MAPT* (*p*-value = 7.57 × 10^−1^, OR = 1.03; *p*-value = 4.77 × 10^−2^, OR = 1.2) loci (Table 5) suggesting that these genetic risk factors seem not to associate with the Italian FTD population.

Conversely, rs2075650 for the *TOMM40*/*APOE* locus revealed a *p*-value = 2.1 × 10^−5^ and OR = 1.65 (Table 5) implying that either this locus might be suggestive for the Italian FTD population or that there is an underlying presence of AD cases within our FTD cohort. Keeping in mind that it had been previously shown that *APOE* genotypes and/or alleles revealed variable associations either with longevity or disability in the Italian population (Bader et al., 1998, Benedetti et al., 2002, Scacchi et al., 1995), we carried forward the analysis of other typical AD genome-wide markers in our study cohort to further investigate the potential presence of AD among the FTD cases; particularly, we analyzed 5 other loci/SNPs that have consistently been associated with AD such as: rs157580 (*TOMM40*/*APOE*), rs11136000 (*CLU*), rs3818361 (*CR1*), rs3851179 (*PICALM*), and rs744373 (*BIN1*) (Harold et al., 2009, Hollingworth et al., 2011, Lambert et al., 2009). Our analysis revealed that none of the markers associated with our study population (*p*-value ∼10^−1^; Table 5) suggesting that most probably the risk of a contamination of AD cases within our FTD cohort is likely to be minimal.

### 3.2 SNPs-to-genes analysis

**Fig. 3:**
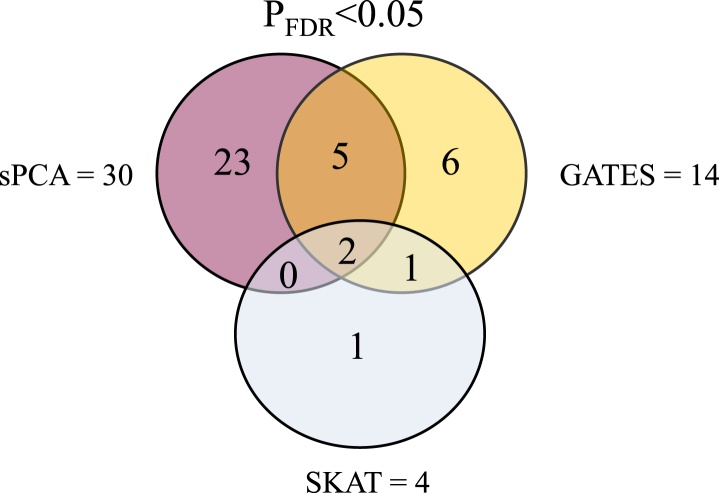
SNPs-to-genes analysis. The Venn diagram shows the number of genes that were significant after FDR correction (*p*-value < 0.05) and their level of overlap across the 3 methods (GATES, sPCA, and SKAT). Abbreviations: FDR, false discovery rate; GATES, extended Simes' procedure; SKAT, sequential kernel machine association test; sPCA, supervised principal components analysis.

To identify genes associated with disease and to replicate the results of our association analysis, we performed GATES, sPCA, and SKAT analyses. Genes with *p*-value < 0.05 after FDR correction are displayed in Fig. 3, Table 6 and described here in the following sections.

#### 3.2.1 GATES analysis

The genes with lowest *p* values were *CEP131*, *ENTHD2,* and *C17orf89* (Table 6), supporting them as suggestive candidates as per association analysis (Table 1). Additional 11 genes resulted significant (Table 6): these were all among the nominally significant genes as per association analysis (Supplementary Table 1). Of note, *HNF1B* that we showed above being a potential sex-specific disease-associated marker (Table 3) was among these 11 genes (Table 6).

#### 3.2.2 sPCA analysis

The sPCA analysis revealed 30 significant genes the first 2 being *CEP131* and *ENTHD2* (supporting the results of the association and the GATES analyses) (Table 6). Comparing the sPCA results with those obtained through GATES, a total of 7 genes (*CEP131*, *ENTHD2*, *TOMM40*, *HNF1B*, *PVRL2*, *SAMD12,* and *C9orf150*) were significant (and confirmed by both methods).

#### 3.2.3 SKAT analysis

The SKAT analysis revealed 4 significant genes (*CEP131*, *ENTHD2*, *C17orf89,* and *QPCT*) (Table 6). *QPCT* was neither in the output of GATES or sPCA nor the association analyses (Table 6 and Supplementary Table 1). SKAT analysis confirmed *CEP131*, *ENTHD2,* and *C17orf89* being significant as per GATES and association analyses.

Taken all together, the 3 analyses (GATES, sPCA, and SKAT) revealed the following: (1) all the significant genes after GATES analysis (*CEP131*, *ENTHD2*, *C17orf89*, *TOMM40*, *HNF1B*, *ZCCHC24*, *PVRL2*, *SLC38A10*, *CCDC12*, *SAMD12*, *GYPC*, *KLHL18*, *RSU1,* and *C9orf150*), belonged to the group of suggestive or nominally significant genes as per association analysis (Table 1 and Suuplementary Table 1); (2) 2 genes (*CEP131* and *ENTHD2*) were consistently identified across the 3 analysis methods (GATES, sPCA, or SKAT) and belonged to the strongly suggestive hits as per association analysis (Table 1); (3) a number of further genes were shared across the GATES and sPCA methods (*TOMM40*, *HNF1B*, *PVRL2*, *SAMD12,* and *C9orf50*), and the GATES and SKAT methods (*C17orf89)*; and (4) a number of genes were unique to each method of analysis: (i) *ZCCHC24*, *SLC38A10*, *CCDC12*, *GYPC*, *KLHL18,* and *RSU1* for the GATES method; (ii) *PRKG1*, *DNAJA4*, *CNTN4*, *MYO10*, *SGCG*, *LPAR3*, *FOXK2*, *NAP5*, *TMEM169*, *TTLL9*, *MUSK*, *SP100*, *SCGB1D4*, *IRF8*, *LARGE*, *PLCB3*, *CDKN3*, *RASGRF1*, *IL1RL1*, *CCDC81*, *WDR45L*, *PTGER3,* and *MDGA2* for the sPCA method; and (iii) *QPCT* for the more conservative SKAT method.

In summary, after the SNPs-to-genes analyses, we confirmed the relevance of the locus on chr 17 (17q25.3), which was shown to be strongly suggestive after association analysis (Table 1). Of note, we did not consistently identify the locus on chr2, 2p16.3, among the significant genes/loci through our SNPs-to-genes analyses as after multiple test corrections for GATES, sPCA, and SKAT no association was evident. This may be due to the fact that because the SNPs-to-genes analyses assessed 2087 SNPs at this locus (2p16.3) and the suggestive SNPs (n = 7) are in strong LD exclusively with each other (see Fig. 2B), this locus was not powerful enough to identify an association. Nevertheless, the suggestive *p* values of the association analysis and the OR of 2.5–2.8 (Table 1) indicate that this locus will benefit from further investigation to gather on its actual role in this population. In total, we identified 8 genes (*CEP131*, *ENTHD2*, *C17orf89*, *TOMM40*, *HNF1B*, *PVRL2*, *SAMD12,* and *C9orf150*) that were suggestive in the association analysis (Table 1 and Supplementary Table 1) and supported by the SNPs-to-genes analysis (Table 6).

### 3.3 GO terms analysis

After having identified a number of DAGs through our association and SNPs-to-genes analyses, we intended to better characterize their functional and biological relevance. Thus, we performed a GO analysis based on a list of 280 DAGs (271 and 9 nonoverlapping genes from the SNP-to-genes and association analyses, respectively; Supplementary Table 5 and Supplementary Fig. 4) specifically looking at how functionally similar GO terms clustered and which were most significant among each category (BP, CC, and MF).

#### 3.3.1 Functional annotation clustering

After the ES (threshold ≥1.3) was calculated for each functional annotation cluster, 3 were found significant with ES of 2.1, 1.8, and 1.7 (Supplementary Table 6 and Supplementary Fig. 5a).

The first cluster (ES = 2.1) includes 37 GO terms. Fourteen of 37 are specific to the brain, particularly, to processes such as brain development, neuronal genesis, maturation and differentiation, and axonogenesis. The remainder terms are more general and point to system development, cell differentiation and projection, as well as cell-cell adhesion. This cluster suggests that our DAGs are involved in systems' development and differentiation into mature structures and it is relevant to note that a robust association with the brain was evident considering the identification of processes related to the development and maturation of neurons and the formation of axons (Supplementary Fig. 5b).

The second cluster (ES = 1.8) includes only 7 GO terms. This cluster reveals enrichment, in general, of genes pointing toward elements of the plasma membrane with receptor-like activity (Supplementary Table 6).

The third cluster (ES = 1.7) includes up to 10 GO terms. It is of relevance that this cluster reveals an enrichment of genes whose products localize to the synapse regulating neurotransmission and plasticity (Supplementary Fig. 5a and 5c).

To support DAVID results a gene-set enrichment analysis for each GO category (BP, CC, and MF) was performed on the totality of the annotated genes ranked by the lowest *p* value from either the GATES, sPCA, or SKAT analysis.

#### 3.3.2 TopGO BP

Considering a minimum of 8 genes to support a GO term enrichment and a significance Kolmogorov-Smirnov *p* value for the “weight01” analysis starting at *p*-value < 0.01, up to 87 GO terms resulted significant for the BP ontology (Supplementary Table 7). The significant terms can be subdivided in 3 groups based on 3 different *p*-value cutoffs. In group 1 (*p*-value < 0.001), there was a total of 16 terms of which 6 directly related to the brain (“synaptic transmission,” “central nervous system development,” “synaptic transmission, glutamatergic,” “dendrite morphogenesis,” “regulation of synaptic transmission, glutamatergic,” and “positive regulation of synaptic transmission, glutamatergic”) replicating the group of BPs highlighted by the third cluster of the DAVID analysis (Supplementary Table 6). There were 3 additional terms related to ion transmembrane transport and “cellular response to epinephrine stimulus,” all suggesting an involvement in neurotransmission processes. In group 2 (0.001 < *p*-value < 0.005), there were 40 GO terms of which 5 directly related to the brain (“striatum development,” “axon guidance,” “long-term synaptic potentiation,” “learning,” and “forebrain neuron differentiation”) and replicated the first cluster of the DAVID analysis (Supplementary Table 6); also, 9 further GO terms here supported the membrane potential regulation process (Supplementary Table 7). Finally, group 3 (0.01 < *p*-value < 0.005) revealed 31 GO terms, 5 of which directly related to the brain (“hypothalamus development,” “negative regulation of axon extension involved in axon guidance,” “regulation of glial cell proliferation,” “cognition,” and “neuron cell-cell adhesion”) and replicated the DAVID analysis results of both clusters 1 and 3 (Supplementary Table 6); in addition, 6 GO terms indicated ion transmembrane transport and 2 regulation of immune response activity (Supplementary Table 7).

#### 3.3.3 TopGO CC

The CC ontology analysis revealed a number of relevant GO terms for each of the 3 groups as per *p*-value cutoff. In group 1, there was a total of 9 GO terms of which 5 (“postsynaptic membrane,” “presynaptic membrane,” “postsynaptic density,” “synapse,” and “dendrite”) directly related to neurotransmission processes and 1 to the “glutamate receptor complex” (Supplementary Table 7). In group 2, there was a total of 9 GO terms 3 of which directly related to the neurons including the “presynaptic active zone” and the “axon” and 2 indicated, respectively, the “voltage-gated calcium channel complex” and the “receptor complex” (Supplementary Table 7). Finally, in group 3 there was a total of 10 GO terms that included the “voltage-gated potassium channel complex,” the “dendritic spine,” and the “MHC class II protein complex”, implying to synaptic transmission and an involvement of transmembrane elements and the immune system (Supplementary Table 7). In summary, this analysis reveals a strong implication of processes happening at the level of the synapsis including neurotransmission in general and at the level of the dendritic spine, resulting supportive of the previous Section 3.3.2 and replicating the third cluster of the DAVID analysis (Supplementary Table 6).

#### 3.3.4 TopGO MF

Further, the MF ontology analysis highlighted several significant GO terms (encompassing groups 1–3). In this respect, the main outcome here is the indication of the process of neurotransmission through terms such as “calmodulin binding,” “ion channel binding,” “ionotropic glutamate receptor activity,” “glutamate receptor binding,” “high voltage-gated calcium channel activity,” “extracellular-glutamate-gated ion channel,” “voltage-gated potassium channel activity,” and “voltage-gated calcium channel activity” (Supplementary Table 7). These results critically highlight that these enrichments are cross-supportive within the MF analysis as well as across the other ontology terms (BP and CC) and all clusters of the DAVID analysis (Supplementary Table 6). As a matter of fact, we see here that activities which imply neuronal membrane receptors, signaling transduction and propagation of electrical signal at the level of the synapses are significantly enriched (Supplementary Table 7).

## 4 Discussion

To the best of our knowledge, this is the first comprehensive genome-wide study on Italian FTD. We performed a classical association analysis and then we further characterized our data set by means of an innovative 3-fold statistical approach that uses *p* values (from the association analysis) or directly the genotyping data to identify genes associated with disease.

Seven SNPs at the 2p16.3 locus were suggestive and the risk alleles showed high OR (>2.5). These SNPs map to the *LOC730100* gene, a long noncoding RNA. Long noncoding RNAs are elements implicated in a number of complex processes that include chromatin stabilization, histone methylation as well as pretranscriptional and post-transcriptional (cis- and trans-) regulation (Mercer et al., 2009). We could not identify a potential disease-associated haplotype at this locus, neither could we verify any effects on transcription in cis for the suggestive SNPs nor were these in LD with any SNP locating to neighboring genes. However, this locus seems to hold importance due to the 2-fold OR value as per association analysis and the vicinity of the *NRXN1* gene. The latter is involved in neurodevelopment and has been shown to carry pathogenic CNVs associated with a spectrum of neurobehavioral and neuropsychiatric disorders (Bena et al., 2013); as such, it cannot currently be fully dismissed as a possible biological reason for association at this locus. We thus suggest that *NRXN1* should be investigated for variability including single nucleotide variants, indels, and CNVs to shed light on its potential role in (Italian) FTD.

Seven SNPs at the 17q25.3locus showed suggestive association with OR > 1.5 for each risk alleles. These SNPs map to 3 genes: *CEP131*, *ENTHD2,* and *C17orf89*. *CEP131* encodes a centrosomal protein of 131 kDa weight, which is part of the centrosomal complex and seems involved in cilia formation and genome stability processes (Staples et al., 2012). *ENTHD2* encodes a protein that localizes to the cytoplasm and seems to be involved in trans-Golgi network vesicular processes (Borner et al., 2012), whereas *C17orf89* is still uncharacterized. Based on this information it is difficult to diagnose a direct impact of these 3 genes on the biology of FTD. Nevertheless, not only this locus and these 3 genes (*CEP131*, *ENTHD2,* and *C17orf89*) have consistently been replicated in our SNPs-to-genes analysis by either method (GATES, sPCA, and SKAT), but also there are a number of reasons to consider the association at this locus of potential biological relevance. First, we verified that the 7 risk alleles of the suggestive SNPs define a haplotype substructure that significantly associated with disease status with an OR = 1.45, and second, each of the risk alleles had significant or suggestive effects on transcription, specifically, causing a decrease of expression of cis genes such as *RFNG*, *AATK*, and *MIR1250*. *RFNG* encodes an N-acetylglucosaminyltransferase for which involvement in neurogenesis and a role in modulating Notch signaling has been previously suggested (Mikami et al., 2001). *AATK* was shown to have a potential role in apoptotic processes in mature neurons (Baker et al., 2001), and even more interestingly, in neuronal differentiation (Baker et al., 2001) or axon outgrowth (Takano et al., 2012). Conversely, a general implication in regulation of transcription and/or gene expression applies to *MIR1250*. Taken together our results suggest that neuronal development, maturation, and axonogenesis, as well as regulation of gene expression might be impacted in the Italian FTD population.

When we tested for association based on gender, we identified 2 SNPs mapping to the *HNF1B* gene being significant in the female FTD population. *HNF1B* encodes a member of the homeodomain-containing family of transcription factors. The product of this gene is expressed in the brain; however, any potential role in the brain has not been described thus far. Rather, *HNF1B* has been suggested being involved in ovarian adenocarcinoma (DeLair et al., 2013, Shen et al., 2013) and renal failure (Musetti et al., 2014). Nevertheless, of particular interest is the fact that variability in this gene seems specifically to associate with conditions affecting women as shown in the current and other studies (DeLair et al., 2013, Shen et al., 2013). All the more, it is relevant to note that this gene is involved in regulation of transcription likewise the *LOC730100* and the *MIR1250* genes, suggesting that regulation of gene expression needs to be considered and further investigated to shed light on its potential contribution to disease etiology.

When we assessed the loci that had previously been associated with FTD, we did not replicate the top SNPs for FTD with TDP-43 pathology (Van Deerlin et al., 2010) neither those as per the international GWAS (Ferrari et al., 2014). This may be due to the fact that, besides the European ancestry, the Mediterranean population might not exactly share the same risk factors as that of Western/Central/North- and American-European extraction. Of note, also the other candidate loci including *MAPT* and *C9orf72* resulted nonsignificant. Particularly, concerning *MAPT*, we also assessed any potential implication of the *MAPT* haplotype in our cohort: we verified that the H1 haplotype did not associate with disease, whereas the H2 haplotype resulted protective, and that the results of our association analysis were independent from the *MAPT* haplotype. Having identified *TOMM40* and *PVRL2* among the significant genes through the SNPs-to-genes analysis, and keeping in mind that these reached nominal significance in our association analysis this indicates that *APOE*, notably an AD locus, needed attention. When we verified association for loci specific to other neurodegenerative diseases, we identified the *TOMM40/APOE* locus reaching *p* value of 2.1 × 10^−5^ (rs2075650; OR = 1.65) in our FTD cohort. However, when we evaluated other AD-GWAS markers for *TOMM40/APOE*, *CLU*, *CR1*, *PICALM,* and *BIN1* there was no association at all. If on one hand we cannot fully exclude a minimal presence of AD cases within our FTD clinical cohort, on the other there is also evidence in the literature of an independent association of the *APOE* locus with FTD, especially involving the *APOE E4* allele (Stevens et al., 1997). This seems to occur also in Italian FTD cases (Rubino et al., 2013, Seripa et al., 2011, Seripa et al., 2012). Particularly, a genetic study showed that *APOE E4* associated with FTD with OR = 2.26 and, as well, variability in *TOMM40* held an *APOE E4*-dependant association with Italian FTD, with OR = 2.11 (Bagnoli et al., 2013). Also, an imaging based study showed that the *APOE E4* allele underlies higher brain vulnerability not only in AD but also in FTD and that other concomitant genetic and/or environmental factor(s) might modulate the detrimental effects of *APOE E4* leading to the different regional vulnerability that is at the basis of the topographical differences in the affected areas in AD (mediotemporal) and FTD (frontotemporal) (Boccardi et al., 2004). Taken all this together and based on our own data, *APOE* might be a locus to be further characterized in the Italian FTD population, and conversely, the risk of a contamination of AD cases within our study cohort, if any, is likely to be minimal.

The classical association analysis was followed by the SNPs-to-genes analysis, an innovative 3-fold statistical approach that uses *p* values (from the association analysis) or directly the genotyping data to identify genes associated with disease. The SNPs-to-genes approach not only is a method to replicate and/or support the results of the association analysis but also provides further insight into the genetics of this disease with a particular focus on genes. This is important because it allows to direct our attention on gene products that can then be taken forward into functional annotation analysis to interpret more comprehensively their biological relevance and to shed light on the biological and/or molecular processes and cellular compartments that might be impacted and influence disease pathogenesis.

Our SNPs-to-genes results, through 3 different methods (GATES, sPCA, and SKAT), supported the data of the association analysis, particularly indicating the 17q25.3 locus as a potential novel locus for the Italian FTD population. These analyses were supportive of the association analysis and contributed to enlarge the list of genes potentially relevant to FTD in the Italian population. We observed that 8 genes indicated by our association analysis were significantly replicated by any of the 3 methods (GATES, sPCA, and SKAT): *CEP131*, *ENTHD2*, *C17orf89*, *TOMM40*, *HNF1B*, *PVRL2*, *SAMD12,* and *C9orf150*. If we have already commented on the first 6 genes of this list, not much can currently be said about the remaining 2 as both, the sterile alpha motif domain containing 12 (*SAMD12*) and the *C9orf150* (or leucine rich adaptor protein 1-like [*LURAP1L*]) genes have not yet been fully characterized, thus it is currently difficult to assess their potential relevance in FTD. All the more, the GATES, sPCA, and SKAT methods identified an overall list of genes suggestive for association with disease (DAGs) in our cohort that we decided to investigate more in depth for their biological meaning and potential implication in the biology of the brain and FTD. In this respect, our enrichment analysis based on an input of 280 genes (derived from the association and the SNPs-to-genes analyses), interestingly indicated that a number of our DAGs are, in fact, implicated in the biology of the brain. Specifically, they revealed that elements regulating neuronal maturation, axonal formation and synapse plasticity, as well as presynaptic and postsynaptic activities (involving neurotransmission), might play a role in the neurobiology of FTD in the Italian population. Of particular interest is the fact that we verified that the risk alleles at the 17q25.3 locus exert an effect on expression, that is, a decrease in expression of cis genes that, in fact, are involved in processes highlighted by our GO terms analysis such as neuronal development, differentiation and maturation, and axonogenesis. All the more, the 2p16.3 locus comprises the *NRXN1* gene that also is involved in neurodevelopment. These data are of interest in that our association and GO terms analysis seem cross-supportive to the extent that they suggest that common mechanisms involving the biology of the neurons and that of the synapses might be implicated in the pathogenesis of FTD in the Italian population. Finally, the enrichment analysis also suggested that processes involving regulation of immune response and MHC class II molecules (and most probably microglial cells) require further study and characterization in the Italian FTD population.

## 5 Conclusions

In summary, the following can be gathered from our study: (1) this is the first genetic study of this size in the Italian FTD population; (2) we identified 2 novel potential loci for FTD; (3) one of the 2 new loci (17q25.3) revealed the existence of a haplotype substructure that significantly associates with disease and likely exerts its effect by affecting expression (decrease) of nearby cis genes; (4) our association analysis results for the 17q25.3 locus were supported by additional SNPs-to-genes analyses performed and validated by means of 3 different statistical methods (GATES, sPCA, and SKAT); (5) genes directly or indirectly highlighted by our analyses, such as *NRXN1*, *RFNG,* and *AATK*, are involved in neuronal development, differentiation and maturation, and axonogenesis processes; (6) our enrichment analyses supported these biological functions revealing significant GO terms that included elements pointing to the biology of the brain, particularly, neurogenesis, neuronal development, differentiation and maturation, as well as the biology of the synapse, including neurotransmission, synapse plasticity, and membrane potential modulation; and (7) our GO terms analysis also showed a potential involvement of microglial cells, thus neuronal pruning (which is part of the process of neurons maturation) and functional brain connectivity (Zhan et al., 2014), as well as an overall involvement of regulation of immune response. Although we need to be cautious about any speculation at present because we did not replicate the same genetic loci, the latter point is of relevance as it might indirectly support the outcome of our international GWAS about an involvement of the immune system in the neurobiology of FTD (Ferrari et al., 2014).

Our study is clearly not conclusive and requires the identified loci and genes to be further studied and replicated especially to discriminate those markers and genes that likely drive the associations and are involved in the susceptible molecular mechanisms potentially at play in the process of pathogenesis of FTD in the Italian population.

## Disclosure statement

The authors have no conflicts of interest to disclose.

## Figures and Tables

**Table 1:** Genome-wide association results for FTD-associated SNPs with *p*-value < 10^−6^

Marker	Chromosome	Position (bp)	Nearest gene	Location	Minor allele	Major allele	Frequency of minor allele	Imputation quality (Rsq)	OR of minor allele (95% CI)	*p* Value
rs906175	17	79,173,462	*CEP131*	Intron 9	T	C	0.48	0.88	1.58 (1.33–1.87)	1.22 × 10^−7^
rs17042852	2	52,600,067	*LOC730100*	Intron 10	C	T	0.04	0.97	2.82 (1.91–4.18)	2.01 × 10^−7^
rs1526678	2	52,635,727	*LOC730100*	intergenic	G	A	0.04	0.99	2.83 (1.91–4.20)	2.19 × 10^−7^
rs17042770	2	52,571,393	*LOC730100*	Intron 8	C	G	0.04	0.96	2.83 (1.91–4.20)	2.22 × 10^−7^
rs2725391	17	79,192,430	*CEP131*	Intron 2	T	C	0.48	0.98	1.52 (1.30–1.78)	2.50 × 10^−7^
rs12621157	2	52,509,876	*LOC730100*	Intron 7	T	G	0.04	0.96	2.75 (1.87–4.07)	3.73 × 10^−7^
rs12622570	2	52,546,301	*LOC730100*	Intron 8	C	G	0.04	0.96	2.76 (1.87–4.09)	3.99 × 10^−7^
rs969413	17	79,195,814	*CEP131*	Intron 1	A	T	0.49	0.95	1.52 (1.29–1.79)	4.26 × 10^−7^
rs2659030	17	79,177,974	*CEP131*	Intron 5	A	G	0.46	0.83	1.56 (1.31–1.86)	4.42 × 10^−7^
rs2255166	17	79,213,562	*C17orf89*	Intron 2	C	T	0.46	0.85	1.55 (1.30–1.84)	6.19 × 10^−7^
rs12619513	2	52,532,874	*LOC730100*	Intron 7	A	G	0.05	0.94	2.53 (1.76–3.65)	6.39 × 10^−7^
rs9319617	17	79,192,446	*CEP131*	Intron 2	C	T	0.48	0.97	0.66 (0.57–0.78)	6.62 × 10^−7^
rs1048775	17	79,202,329	*ENTHD2*	3′-UTR	G	C	0.50	0.91	0.66 (0.56–0.78)	8.04 × 10^−7^
rs12614311	2	52,521,716	*LOC730100*	Intron 7	T	C	0.06	0.81	2.55 (1.75–3.71)	8.83 × 10^−7^

To retrieve information about SNPs and their genomic context (the nearest gene and location) we used the hg18 (NCBI 36) assembly. We performed a logistic regression of FTD status on SNP dosage levels adjusting for sex and the first 4 principal components. The SNPs are ranked by *p* value.

Key: CI, confidence interval; FTD, frontotemporal dementia; OR, odds ratio; SNPs, single nucleotide polymorphisms.

**Table 2:** Haplotype analysis at the 17q25.3 locus

Locus	Haplotype	Frequency	Frequency FTD case	Frequency controls	OR (95% CI)	*p* Value
rs906175, rs2659030, rs9912789, rs9896850, rs2725391, rs9319617, rs12939525, rs8073077, rs969413, rs1048775, rs2255166, rs2659005	(1) CGTTCCCTTGTC	0.43	0.39	0.49	0.695 (0.59–0.82)	7.42 × 10^−6^
(2) TACCTTTCACCT	0.43	0.52	0.42	1.42 (1.20–1.65)	1.60 × 10^−5^
(3) CGCCCCTCTGTC	0.02	0.02	0.03	0.71 (0.41–1.23)	21.7 × 10^−1^
(4) TACCTTTCACCC	0.02	0.03	0.02	1.35 (0.79–2.31)	2.92 × 10^−1^
(5) TACCTTTCACTT	0.02	0.02	0.02	1.31 (0.71–2.40)	3.83 × 10^−1^
(6) CGCCCTTCTGTC	0.02	0.03	0.02	1.005 (0.77–1.32)	9.73 × 10^−2^
rs906175,rs2659030, rs2725391, rs969413, rs1048775, rs9319617, rs2255166	(1) CGCTGCT	0.47	0.42	0.52	0.67 (0.58–0.79)	8.30 × 10^−7^
(2) TATACTC	0.45	0.53	0.43	1.45 (1.23–1.69)	4.39 × 10^−6^
(3) CGCACCC	0.01	0.01	0.01	0.56 (0.25–1.24)	1.50 × 10^−1^
(4) TATACTT	0.02	0.02	0.02	1.37 (0.77–2.40)	2.86 × 10^−1^
(5) CGCTGTT	0.02	0.02	0.02	0.92 (0.55–1.55)	7.56 × 10^−1^

Logistic regression results for haplotypes constructed using 12 and 7 SNPs mapping to the long arm of chr 17 (17q25.3 locus), adjusting for sex and the first 4 principal components. For each haplotype, we reported the frequency in FTD cases and controls.

Key: CI, confidence interval; FTD, frontotemporal dementia; OR, odds ratio; SNPs, single nucleotide polymorphisms.

**Table 3:** Gender stratified analysis

SNP	*p*-Value females	OR (CI 95%) females	*p*-Value males	OR (CI 95%) males	*p*-Value int	OR (CI 95%) int	Coded allele	Gene	Function
rs3110642	3.11 × 10^−6^	2.15 (1.56–2.96)	5.80 × 10^−2^	1.32 (0.99–1.76)	0.022	1.62 (1.08–2.53)	C	*HNF1B*	Intron
rs3110643	4.55 × 10^−6^	2.12 (1.54–2.92)	5.24 × 10^−2^	1.33 (0.997–1.78)	0.027	1.59 (1.05–2.50)	C	*HNF1B*	Intron
rs3110648	1.51 × 10^−5^	1.96 (1.44–2.65)	2.18 × 10^−2^	1.38 (1.05–1.82)	0.077	1.42 (0.96–2.17)	G	*HNF1B*	Intron
rs9912789	2.25 × 10^−5^	0.61 (0.48–0.77)	8.02 × 10^−3^	0.74 (0.59–0.92)	0.259	0.83 (0.60–1.15)	T	*CEP131*	Intron
rs2659030	3.41 × 10^−5^	1.61 (1.29–2.02)	2.76 × 10^−3^	1.41 (1.13–1.78)	0.451	1.14 (0.82–1.54)	A	*CEP131*	Intron
rs906175	3.43 × 10^−5^	1.61 (1.29–2.02)	3.96 × 10^−4^	1.51 (1.20–1.89)	0.728	1.07 (0.77–1.45)	T	*CEP131*	Intron
rs9319617	4.16 × 10^−5^	0.62 (0.50–0.78)	1.80 × 10^−3^	0.70 (0.56–0.89)	0.501	0.89 (0.65–1.23)	C	*CEP131*	Intron
rs9896850	7.56 × 10^−5^	0.63 (0.50–0.79)	2.36 × 10^−3^	0.70 (0.56–0.88)	0.519	0.89 (0.65–1.24)	T	*CEP131*	Intron

We reported the logistic regression results for females (n = 637) and for males (n = 821) and the gender × SNP interaction analysis for the total sample (n = 1458) with odds ratio (OR) = OR (female)/OR (male). For each SNP, we reported the coded allele associated to odds ratio and the relative mapped gene and function.

Key: CI, confidence interval; OR, odds ratio; SNP, single-nucleotide polymorphism.

**Table 4:** Case and/or control *MAPT* haplotype association analysis

MAPT haplotype^a^	FTD (n = 516)	Controls (n = 909)	OR (95% CI)	*p* Value
H1/H1	302 (58.5%)	503 (55.3%)	1.20 (0.96–1.51)	0.107
H1/H2	194 (37.1%)	349 (38.4%)	0.93 (0.74–1.18)	0.566
H2/H2	29 (3.90%)	57 (6.30%)	0.54 (0.32–0.92)	0.025

For each *MAPT* haplotype, we reported the frequencies (numbers and %) in FTD cases and controls. In both analyses, the logistic regressions are corrected for sex and the first 4 principal components.

Key: CI, confidence interval; FTD, frontotemporal dementia, MAPT, microtubule-associated protein tau.

a Sixteen cases and 17 controls had missing values.

**Table 5:** Genome-wide association results for published SNPs in FTD and other neurodegenerative diseases

	Gene	Minor allele	Major allele	Frequency of minor allele	OR of minor allele (95% CI)	*p* Value	Imputation quality
Marker^a^							
rs1990622	*TMEM106B*	G	A	0.39	0.92 (0.78–1.08)	3.19 × 10^−1^	0.100
rs6966915	*TMEM106B*	T	C	0.39	0.93 (0.79–1.09)	3.73 × 10^−1^	0.998
rs1020004	*TMEM106B*	C	T	0.28	0.95 (0.80–1.14)	5.96 × 10^−1^	0.999
rs302652	*RAB38*	A	T	0.25	0.97 (0.81–1.16)	7.26 × 10^−1^	0.975
rs16913634	*RAB38/CTSC*	A	G	0.11	1.07 (0.82–1.38)	6.23 × 10^−1^	0.882
rs9268877	*HLA-DRA/HLA-DRB5*	A	G	0.42	0.91 (0.77–1.07)	2.54 × 10^−1^	0.987
rs9268856	*HLA-DRA/HLA-DRB5*	A	C	0.19	0.87 (0.71–1.07)	1.82 × 10^−1^	0.988
rs1980493	*BTNL2*	C	T	0.10	0.82 (0.60–1.11)	1.97 × 10^−1^	0.901
rs3849942	*C9orf72/MOB3B*	T	C	0.29	1.21 (1.02–1.43)	3.00 × 10^−2^	1.000
rs242557	*MAPT*	A	G	0.33	1.03 (0.87–1.22)	7.57 × 10^−1^	0.995
rs8070723	*MAPT*	G	A	0.25	0.83 (0.69–1.00)	4.77 × 10^−2^	0.999
rs2075650	*TOMM40/APOE*	G	A	0.12	1.65 (1.31–2.08)	2.10 × 10^−5^	0.996
Marker^b^							
rs157580	*TOMM40/APOE*	G	A	0.39	0.87 (0.74–1.02)	9.17 × 10^−2^	0.993
rs11136000	*CLU*	T	C	0.35	0.86 (0.68–1.08)	1.94 × 10^−1^	0.502
rs3818361	*CR1*	A	G	0.20	1.01 (0.83–1.23)	9.47 × 10^−1^	0.997
rs3851179	*PICALM*	T	C	0.37	1.00 (0.85–1.18)	9.93 × 10^−1^	0.998
rs744373	*BIN1*	G	A	0.28	0.97 (0.81–1.15)	6.98 × 10^−1^	0.986

Key: AD, Alzheimer's disease; ALS; amyotrophic lateral sclerosis; FTD, frontotemporal dementia; GWAS; genome-wide association studies; SNP, single-nucleotide polymorphism.

a Results for SNPs associated with FTD (*TMEM106B*, *RAB38* and *CTSC*, *HLA-DRA*/*DRB5* and *BTNL2*); FTD-ALS (*C9orf72*/*MOB3B*); FTD-17 (*MAPT*), and AD (*TOMM40*/*APOE*).

b Analysis for AD-specific loci highlighted by major AD-GWAS. We reported the results of the logistic regression under an additive model on SNP dosage levels adjusting for sex and the first 4 principal components. To retrieve information about SNPs and their genomic context (the nearest gene), we used the hg18 (NCBI 36) assembly.

**Table 6:** SNPs-to-genes analysis

Type of analysis	Gene	Chromosome	No. snps	No. noprun	*p* Value	*p*(B&H)
GATES	*CEP131*	17	35	19	8.71 × 10^−8^	0.001469158
*ENTHD2*	17	3	3	6.19 × 10^−7^	0.004264162
*C17orf89*	17	3	3	7.59 × 10^−7^	0.004264162
*TOMM40*	19	7	7	2.61 × 10^−6^	0.010990904
*HNF1B*	17	49	44	3.86 × 10^−6^	0.013022048
*ZCCHC24*	10	71	65	7.17 × 10^−6^	0.020079536
*PVRL2*	19	35	27	8.33 × 10^−6^	0.020079536
*SLC38A10*	17	45	39	1.30 × 10^−5^	0.026345165
*CCDC12*	3	24	21	1.41 × 10^−5^	0.026345165
*SAMD12*	8	561	448	1.66 × 10^−5^	0.028043171
*GYPC*	2	82	75	2.69 × 10^−5^	0.039120914
*KLHL18*	3	22	22	2.78 × 10^−5^	0.039120914
*RSU1*	10	261	202	3.04 × 10^−5^	0.039225533
*C9orf150*	9	58	50	3.26 × 10^−5^	0.039225533
sPCA	*CEP131*	17	35	5	4.11 × 10^−8^	0.000649963
*ENTHD2*	17	3	2	1.43 × 10^−7^	0.001006946
*PRKG1*	10	1454	226	1.91 × 10^−7^	0.001006946
*PVRL2*	19	35	7	6.85 × 10^−7^	0.002710751
*C9orf150*	9	58	10	2.92 × 10^−6^	0.009232674
*DNAJA4*	15	22	4	3.55 × 10^−6^	0.009367238
*CNTN4*	3	1300	342	4.56 × 10^−6^	0.010301712
*HNF1B*	17	49	12	5.44 × 10^−6^	0.010760416
*TOMM40*	19	7	2	7.21 × 10^−6^	0.012686542
*MYO10*	5	287	54	8.66 × 10^−6^	0.013705096
*SGCG*	13	262	34	1.67 × 10^−5^	0.022891771
*LPAR3*	1	85	16	1.74 × 10^−5^	0.022891771
*FOXK2*	17	55	10	2.08 × 10^−5^	0.024754692
*NAP5*	2	827	129	2.19 × 10^−5^	0.024754692
*TMEM169*	2	24	6	2.66 × 10^−5^	0.028034957
*TTLL9*	20	45	9	2.92 × 10^−5^	0.028884881
*MUSK*	9	136	24	3.17 × 10^−5^	0.029550084
*SP100*	2	160	50	3.53 × 10^−5^	0.031011342
*SCGB1D4*	11	13	3	4.62 × 10^−5^	0.038479061
*IRF8*	16	69	11	5.20 × 10^−5^	0.041181866
*LARGE*	22	831	169	6.02 × 10^−5^	0.045098118
*PLCB3*	11	8	2	6.40 × 10^−5^	0.045098118
*CDKN3*	14	16	5	6.65 × 10^−5^	0.045098118
*RASGRF1*	15	115	24	6.84 × 10^−5^	0.045098118
*IL1RL1*	2	83	15	8.17 × 10^−5^	0.047840935
*CCDC81*	11	76	14	8.29 × 10^−5^	0.047840935
*WDR45L*	17	41	9	8.34 × 10^−5^	0.047840935
*PTGER3*	1	193	35	8.46 × 10^−5^	0.047840935
*MDGA2*	14	933	168	8.88 × 10^−5^	0.048410287
*SAMD12*	8	561	78	9.17 × 10^−5^	0.048410287
SKAT	*CEP131*	17	35	35	8.35 × 10^−10^	0.000014
*ENTHD2*	17	3	3	1.05 × 10^−7^	0.000651
*C17orf89*	17	3	3	1.15 × 10^−7^	0.000651
*QPCT*	2	34	34	2.39 × 10^−6^	0.010138

Genes with *p*-value < 0.05 after FDR correction by GATES, sPCA, and SKAT methods.

Key: FDR, false discovery rate; GATES, extended Simes' procedure; No. noprun, number of SNPs not pruning by the method; No. snps, number of SNPs cover by gene; *p*(B&H), *p* value after FDR correction with the Benjamini & Hochberg procedure; *p* value, raw *p* value; SKAT, sequential kernel machine association test; sPCA, supervised principal component analysis.
