# Supplementary material for: A genome-wide screening and SNPs-to-genes approach to identify novel genetic risk factors associated with frontotemporal dementia
Source: Neurobiol Aging. 2015 Oct;36(10):2904.e13–26. doi: 10.1016/j.neurobiolaging.2015.06.005 (PMC4706156; doi:10.1016/j.neurobiolaging.2015.06.005)
Supplement: Supplementary Figs. 1–5 and Supplementary Tables 1–8 [file mmc1.pdf]

Supporting Information

S1 Fig. Quantile-quantile (q-q) plot.

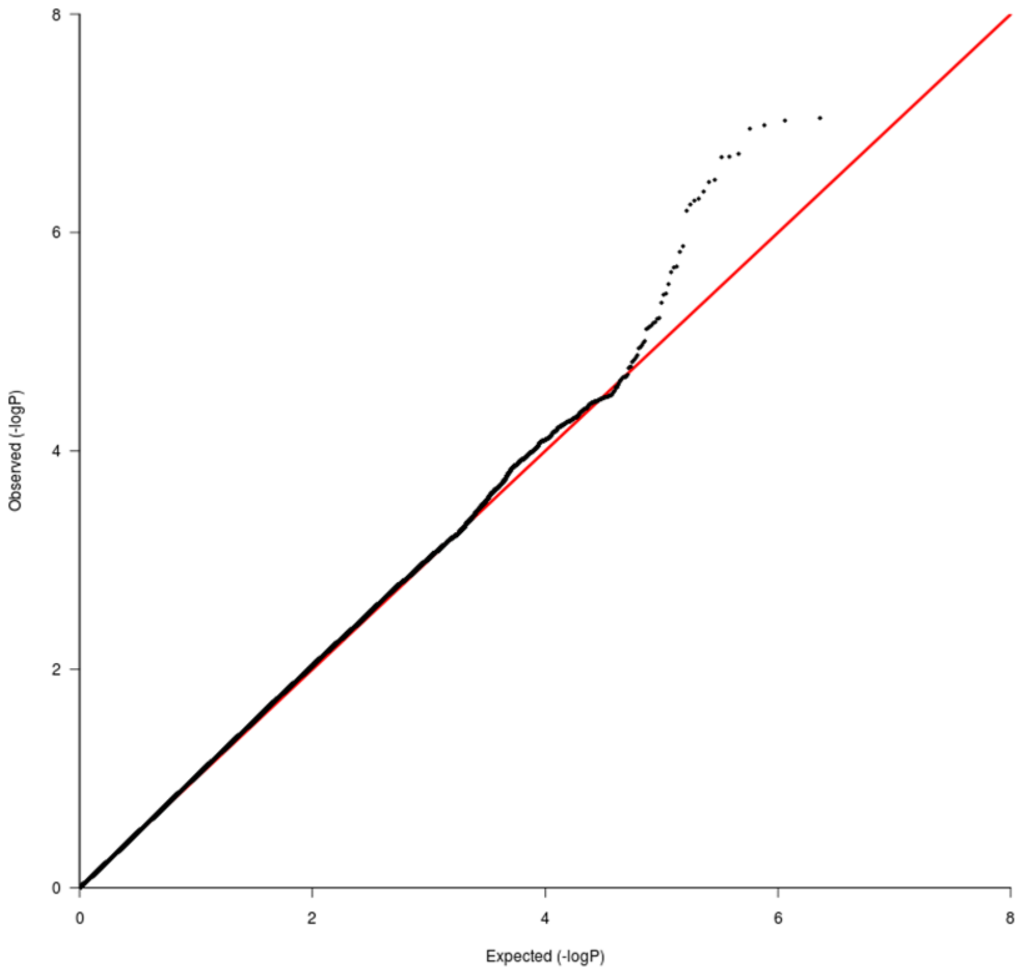

The q-q plot for expected (x-axis) vs. observed (y-axis) p-values is shown. Lambda value was = 1.095

S2 Fig. Loss of heterozygosity (LOH) analysis.

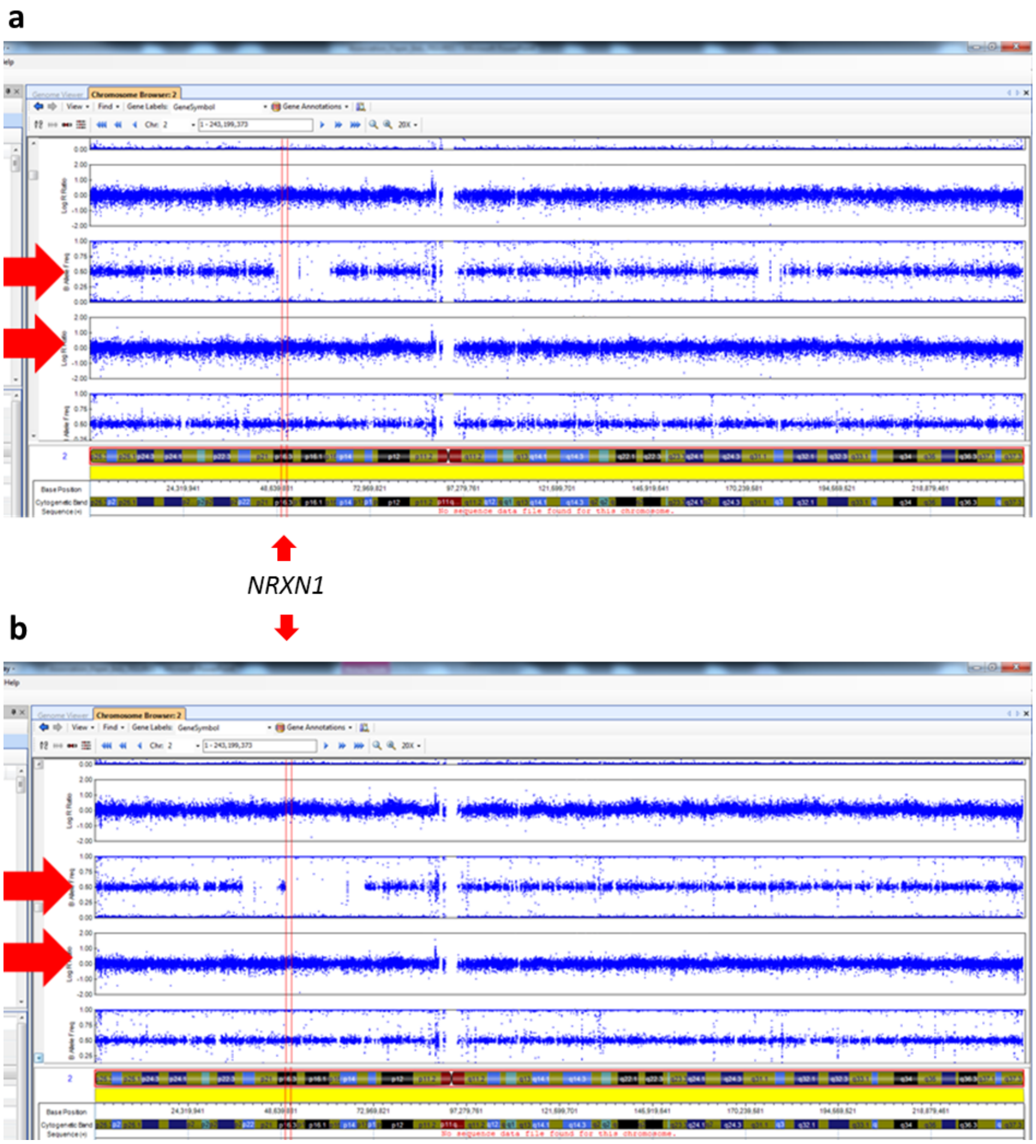

Visual analysis of the distribution of the SNPs at the *NRXN1* locus through the Illumina Genome Viewer (IGV) in Genome Studio reveals no evidence for CNVs within *NRXN1* in our sample set. An example for 2 different FTD cases (**a** and **b**) is shown.

S3 Fig. eQTL analysis.

a *RFNG*

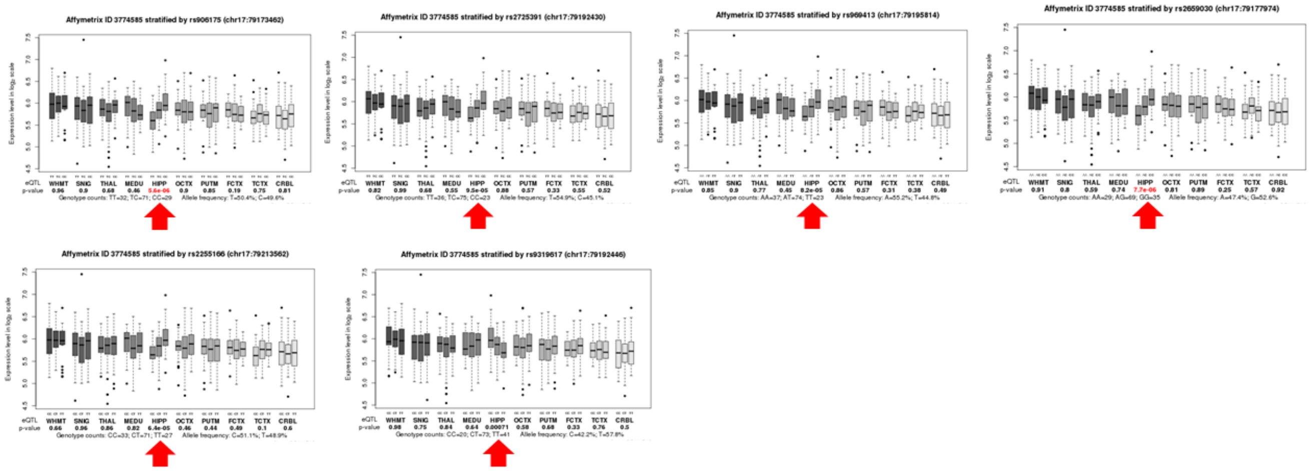

b *AATK & MIR1250*

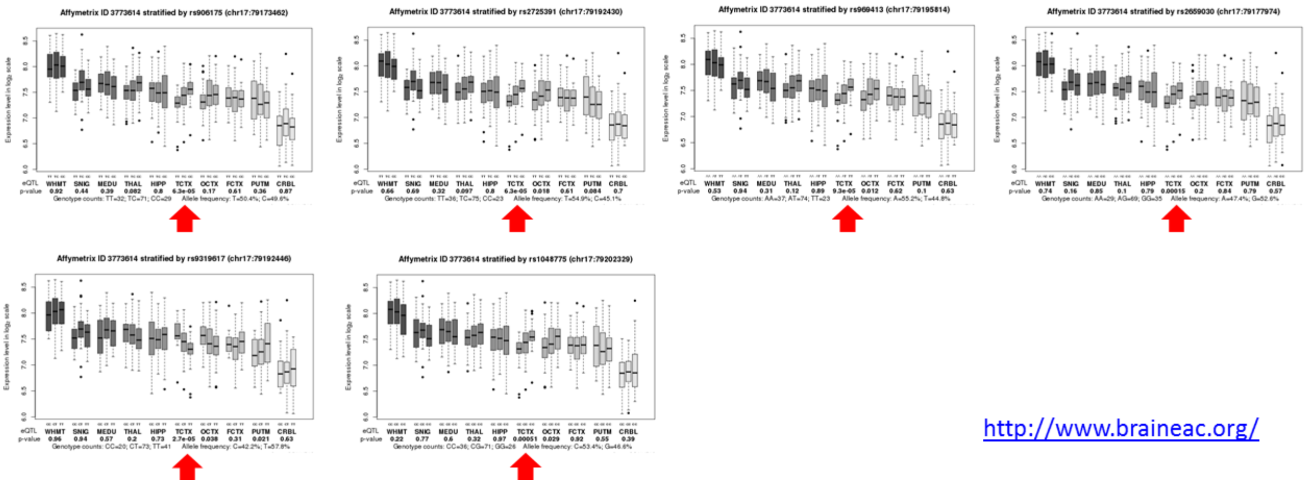

http://www.braineac.org/

a) Box plots for eQTL at the 17q25.3locus. Suggestive SNPs at this locus exerted either significant or suggestive effect on expression in the nearby *cis* gene *RFNG*. Each risk allele causes a decrease of its transcript levels. **b**) Box plots for eQTL at the 17q25.3locus. Suggestive SNPs at this locus exerted suggestive effect on expression in the nearby *cis* genes *AATK & MIR1250*. Each risk allele causes a decrease of their transcript levels. The box plots were obtained from http://www.braineac.org/.

S4 Fig. **SNPs-to-genes analysis**.

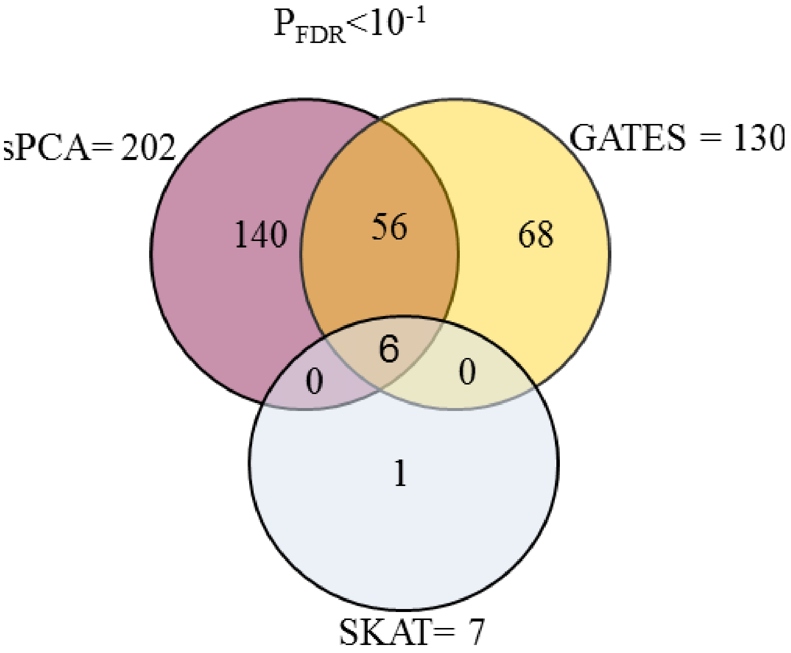

The Venn diagram shows the number of genes that were suggestive after FDR correction (p-value < 0.10) and their level of overlap across the three methods (GATES, sPCA and SKAT).

S5 Fig. **Graphic representation of functional annotation clustering (ES > 1.3)**.

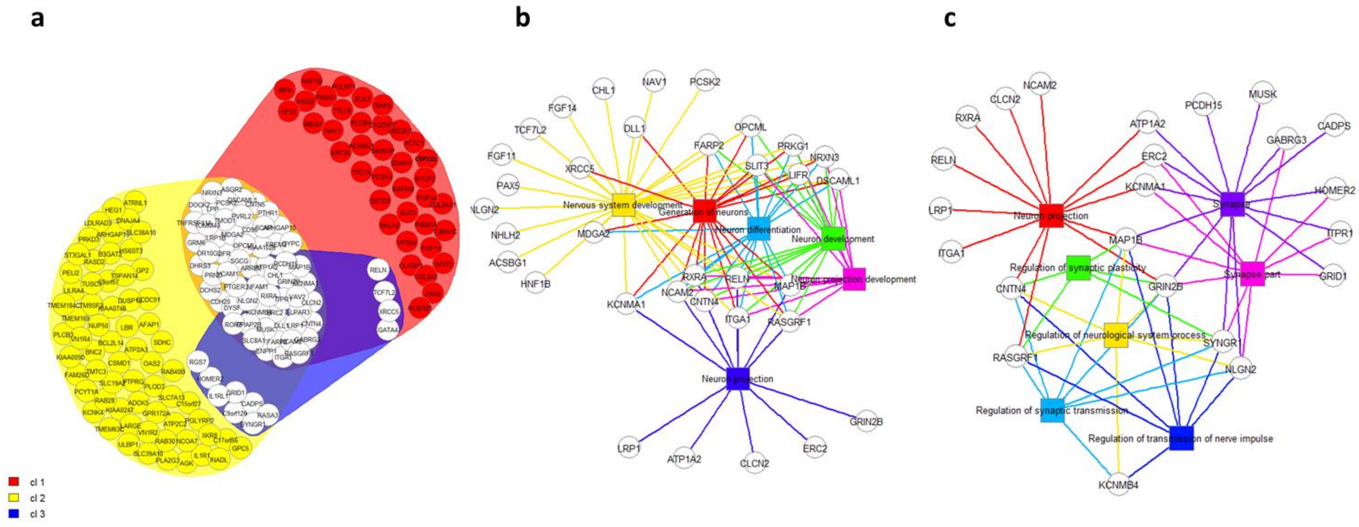

a) Visual distribution of the genes (DAGs) contributing to the three significant clusters. The level of gene overlap across different clusters is depicted. **b**) Visual representation of cluster 1 highlighting the brain-specific ontology terms (squares) and the relative genes (spheres). **c**) Visual representation of cluster 3 highlighting the brain-specific ontology terms (squares) and the relative genes (spheres).

S1 Table. Genome wide association results for FTD-associated SNPs with P-value $< 10^{-4}$.

Marker	Chr	Bp hg19	Al1	A freq1	Or al1	Al2	A freq2	Or al2	Std err	Wald chisq	Pvalue	Lr chisq	Lr pval	R sqr	Gene name
rs906175	17	79173462	C	0.52	0.63	T	0.48	1.58	0.09	27.99	1.22E-07	28.59	8.94E-08	0.88	*CEP131*
rs17042852	2	52600067	T	0.96	0.35	C	0.04	2.82	0.20	27.02	2.01E-07	28.49	9.43E-08	0.97	*LOC730100*
rs1526678	2	52635727	A	0.96	0.35	G	0.04	2.83	0.20	26.86	2.19E-07	28.15	1.12E-07	0.99	*LOC730100*
rs17042770	2	52571393	G	0.96	0.35	C	0.04	2.83	0.20	26.83	2.22E-07	28.30	1.04E-07	0.96	*LOC730100*
rs2725391	17	79192430	T	0.48	1.52	C	0.52	0.66	0.08	26.60	2.50E-07	27.13	1.91E-07	0.98	*CEP131*
rs12621157	2	52509876	G	0.96	0.36	T	0.04	2.75	0.20	25.83	3.73E-07	27.01	2.02E-07	0.96	*LOC730100*
rs12622570	2	52546301	G	0.96	0.36	C	0.04	2.76	0.20	25.70	3.99E-07	27.00	2.04E-07	0.96	*LOC730100*
rs969413	17	79195814	T	0.51	0.66	A	0.49	1.52	0.08	25.57	4.26E-07	26.07	3.30E-07	0.95	*CEP131*
rs2659030	17	79177974	G	0.54	0.64	A	0.46	1.56	0.09	25.50	4.42E-07	25.98	3.44E-07	0.83	*CEP131*
rs2255166	17	79213562	T	0.54	0.65	C	0.46	1.55	0.09	24.85	6.19E-07	25.30	4.90E-07	0.85	*C17orf89*
rs12619513	2	52532874	G	0.95	0.40	A	0.05	2.53	0.19	24.79	6.39E-07	25.59	4.22E-07	0.94	*LOC730100*
rs9319617	17	79192446	T	0.52	1.51	C	0.48	0.66	0.08	24.72	6.62E-07	25.22	5.10E-07	0.97	*CEP131*
rs1048775	17	79202329	C	0.50	1.52	G	0.50	0.66	0.09	24.35	8.04E-07	24.82	6.31E-07	0.91	*ENTHD2*
rs12614311	2	52521716	C	0.94	0.39	T	0.06	2.55	0.19	24.17	8.83E-07	25.07	5.54E-07	0.81	*LOC730100*
rs9896850	17	79191726	C	0.54	1.50	T	0.46	0.67	0.09	22.91	1.70E-06	23.38	1.33E-06	0.94	*CEP131*
rs2659005	17	79218714	T	0.49	1.51	C	0.51	0.66	0.09	22.75	1.85E-06	23.14	1.51E-06	0.89	*SLC38A10*
rs3110648	17	36071034	T	0.82	0.61	G	0.18	1.63	0.10	22.44	2.17E-06	22.51	2.09E-06	0.99	*HNF1B*
rs9912789	17	79178196	C	0.54	1.51	T	0.46	0.66	0.09	22.12	2.57E-06	22.55	2.05E-06	0.88	*CEP131*
rs11099660	4	148513620	C	0.70	1.57	T	0.30	0.64	0.10	21.57	3.42E-06	22.33	2.30E-06	0.92	*TMEM184C*
rs3110643	17	36073492	T	0.84	0.61	C	0.16	1.65	0.11	21.44	3.66E-06	21.46	3.62E-06	1.00	*HNF1B*
rs12939525	17	79194005	T	0.55	1.47	C	0.45	0.68	0.08	21.42	3.68E-06	21.84	2.96E-06	0.97	*CEP131*
rs3110642	17	36073620	T	0.84	0.61	C	0.16	1.64	0.11	21.40	3.73E-06	21.42	3.70E-06	1.00	*HNF1B*
rs4980079	10	81194219	C	0.55	0.68	T	0.45	1.46	0.08	20.78	5.15E-06	21.08	4.40E-06	0.98	*ZCCHC24*
rs7083790	10	81194629	C	0.52	0.69	G	0.48	1.45	0.08	20.15	7.17E-06	20.47	6.06E-06	0.99	*ZCCHC24*
rs6857	19	45392254	C	0.87	0.59	T	0.13	1.70	0.12	20.06	7.50E-06	20.17	7.09E-06	0.92	*PVRL2*
rs8073077	17	79195021	C	0.56	1.44	T	0.44	0.69	0.08	20.06	7.51E-06	20.43	6.18E-06	0.99	*CEP131*
rs4980081	10	81196683	C	0.52	0.69	G	0.48	1.45	0.08	19.99	7.80E-06	20.30	6.61E-06	0.99	*ZCCHC24*
rs4980083	10	81197177	G	0.52	0.69	T	0.48	1.45	0.08	19.95	7.94E-06	20.27	6.73E-06	0.99	*ZCCHC24*
rs7086488	10	81200432	G	0.52	0.68	A	0.48	1.46	0.09	19.81	8.54E-06	20.13	7.25E-06	0.93	*ZCCHC24*
rs9643128	8	119445101	T	0.64	0.68	C	0.36	1.47	0.09	19.38	1.07E-05	19.54	9.86E-06	0.89	*SAMD12*
rs10172083	2	40795847	G	0.95	0.42	T	0.05	2.36	0.20	19.31	1.11E-05	19.47	1.02E-05	0.85	*SLC8A1*
rs1495158	4	11517692	T	0.72	0.67	G	0.28	1.50	0.09	19.27	1.13E-05	19.36	1.08E-05	0.93	
rs9997161	4	11516724	G	0.72	0.67	A	0.28	1.48	0.09	19.17	1.20E-05	19.26	1.14E-05	0.96	
rs13092573	3	46988561	C	0.69	0.66	T	0.31	1.51	0.09	19.10	1.24E-05	19.28	1.13E-05	0.86	*CCDC12*
rs10190079	2	52514065	A	0.83	0.63	G	0.17	1.59	0.11	18.95	1.34E-05	18.97	1.33E-05	0.97	
rs295433	3	47362080	C	0.67	0.67	T	0.33	1.49	0.09	18.63	1.58E-05	18.81	1.45E-05	0.84	*KLHL18*
rs7893556	10	16771273	C	0.51	1.41	T	0.49	0.71	0.08	18.42	1.78E-05	18.69	1.54E-05	0.99	*RSU1*
rs12467265	2	40791633	C	0.95	0.44	T	0.05	2.28	0.19	18.35	1.84E-05	18.51	1.69E-05	0.84	*SLC8A1*
rs12467166	2	40791221	G	0.95	0.44	A	0.05	2.28	0.19	18.34	1.85E-05	18.50	1.70E-05	0.85	*SLC8A1*
rs7599354	2	127443771	T	0.70	1.48	G	0.30	0.67	0.09	18.18	2.02E-05	18.75	1.49E-05	0.93	*GYPC*
rs2075650	19	45395619	A	0.88	0.61	G	0.12	1.65	0.12	18.10	2.10E-05	18.18	2.01E-05	1.00	*TOMM40*

Marker	Chr	Bp hg19	Al1	A freq1	Or al1	Al2	A freq2	Or al2	Std err	Wald chisq	Pvalue	Lr chisq	Lr pval	R sqr	Gene name
rs17026205	2	40787615	C	0.95	0.46	G	0.05	2.20	0.19	17.96	2.26E-05	18.12	2.08E-05	0.98	*SLC8A1*
rs12712694	2	40788070	C	0.95	0.46	T	0.05	2.20	0.19	17.94	2.28E-05	18.10	2.10E-05	0.98	*SLC8A1*
rs13431385	2	40787911	C	0.95	0.46	T	0.05	2.20	0.19	17.94	2.28E-05	18.10	2.10E-05	0.98	*SLC8A1*
rs12712695	2	40788108	G	0.95	0.46	C	0.05	2.20	0.19	17.93	2.29E-05	18.09	2.11E-05	0.98	*SLC8A1*
rs10788470	10	87687364	T	0.55	0.71	C	0.45	1.41	0.08	17.76	2.51E-05	17.98	2.23E-05	1.00	*GRID1*
rs13251354	8	119437946	T	0.69	0.69	C	0.31	1.46	0.09	17.59	2.75E-05	17.69	2.61E-05	0.91	*SAMD12*
rs11994945	8	119437743	C	0.69	0.69	T	0.31	1.46	0.09	17.58	2.75E-05	17.69	2.61E-05	0.91	*SAMD12*
rs13253470	8	119438012	C	0.69	0.69	T	0.31	1.46	0.09	17.58	2.75E-05	17.68	2.61E-05	0.91	*SAMD12*
rs7924131	10	87694876	C	0.56	0.71	G	0.45	1.41	0.08	17.57	2.77E-05	17.79	2.47E-05	1.00	*GRID1*
rs9309056	2	40783406	T	0.90	0.58	C	0.10	1.74	0.13	17.51	2.86E-05	17.46	2.93E-05	0.97	*SLC8A1*
rs16929633	9	12823504	C	0.88	0.61	T	0.12	1.64	0.12	17.42	2.99E-05	17.40	3.03E-05	1.00	*LURAP1L*
rs16929631	9	12822891	G	0.88	0.61	A	0.12	1.64	0.12	17.38	3.06E-05	17.36	3.10E-05	1.00	*LURAP1L*
rs7923344	10	87695016	G	0.56	0.71	A	0.44	1.41	0.08	17.34	3.12E-05	17.55	2.80E-05	1.00	*GRID1*
rs13427887	2	40786826	G	0.95	0.47	A	0.05	2.15	0.18	17.33	3.15E-05	17.51	2.86E-05	1.00	*SLC8A1*
rs13040963	20	62027516	A	0.77	1.53	T	0.23	0.65	0.10	17.32	3.16E-05	17.95	2.26E-05	0.93	
rs6122435	20	62022805	T	0.76	1.53	C	0.24	0.65	0.10	17.23	3.31E-05	17.87	2.37E-05	0.91	
rs13390203	2	40786740	T	0.95	0.47	A	0.05	2.14	0.18	17.19	3.39E-05	17.36	3.09E-05	1.00	*SLC8A1*
rs10172665	2	40790377	G	0.95	0.45	A	0.05	2.20	0.19	17.17	3.41E-05	17.30	3.20E-05	0.92	*SLC8A1*
rs10169639	2	40789912	C	0.95	0.46	T	0.05	2.20	0.19	17.17	3.42E-05	17.29	3.21E-05	0.92	*SLC8A1*
rs16867695	5	38752991	G	0.90	0.59	A	0.10	1.69	0.13	17.17	3.43E-05	17.11	3.53E-05	1.00	
rs10195941	2	40790362	A	0.95	0.46	C	0.05	2.20	0.19	17.16	3.43E-05	17.28	3.22E-05	0.92	*SLC8A1*
rs2042019	2	40789687	A	0.95	0.46	C	0.05	2.20	0.19	17.15	3.45E-05	17.28	3.23E-05	0.92	*SLC8A1*
rs2042020	2	40789577	C	0.95	0.46	T	0.05	2.20	0.19	17.15	3.46E-05	17.27	3.24E-05	0.92	*SLC8A1*
rs16867658	5	38727345	C	0.90	0.59	A	0.10	1.69	0.13	17.13	3.48E-05	17.08	3.59E-05	1.00	
rs10857968	1	109367944	A	0.48	0.73	G	0.52	1.38	0.08	17.11	3.53E-05	17.36	3.09E-05	0.99	*AKNAD1*
rs10055279	5	38725043	A	0.90	0.59	C	0.10	1.69	0.13	17.08	3.59E-05	17.02	3.70E-05	1.00	
rs10070418	5	38724924	C	0.90	0.59	G	0.10	1.69	0.13	17.08	3.59E-05	17.02	3.70E-05	1.00	
rs1338526	1	109368249	C	0.48	0.73	G	0.52	1.38	0.08	17.07	3.61E-05	17.32	3.16E-05	0.99	*AKNAD1*
rs4952420	2	40786196	G	0.95	0.47	A	0.05	2.14	0.18	17.06	3.61E-05	17.24	3.30E-05	0.98	*SLC8A1*
rs4952419	2	40786134	C	0.95	0.47	T	0.05	2.14	0.18	17.06	3.62E-05	17.23	3.31E-05	0.98	*SLC8A1*
rs10198424	2	40796445	A	0.97	0.37	C	0.03	2.68	0.24	17.06	3.62E-05	17.08	3.59E-05	0.82	*SLC8A1*
rs11642286	16	85921609	A	0.96	2.86	G	0.04	0.35	0.25	17.06	3.63E-05	20.06	7.51E-06	0.87	
rs1145789	6	91623879	T	0.79	0.66	C	0.21	1.51	0.10	17.05	3.64E-05	17.05	3.64E-05	1.00	
rs7893906	10	87697526	T	0.56	0.71	C	0.44	1.40	0.08	17.04	3.65E-05	17.24	3.29E-05	0.99	*GRID1*
rs13401944	2	40786575	A	0.95	0.47	G	0.05	2.13	0.18	17.04	3.66E-05	17.21	3.35E-05	1.00	*SLC8A1*
rs13402063	2	40786694	A	0.95	0.47	G	0.05	2.13	0.18	17.04	3.67E-05	17.20	3.36E-05	1.00	*SLC8A1*
rs2042021	2	40789428	C	0.95	0.46	T	0.05	2.16	0.19	17.00	3.74E-05	17.12	3.50E-05	0.94	*SLC8A1*
rs2042022	2	40789395	G	0.95	0.46	A	0.05	2.16	0.19	17.00	3.75E-05	17.12	3.51E-05	0.95	*SLC8A1*
rs17026229	2	40788939	G	0.95	0.46	T	0.05	2.16	0.19	16.99	3.76E-05	17.11	3.52E-05	0.95	*SLC8A1*
rs10178831	2	40788917	T	0.95	0.46	A	0.05	2.16	0.19	16.99	3.76E-05	17.11	3.52E-05	0.95	*SLC8A1*
rs8000919	13	103747614	C	0.49	1.39	A	0.51	0.72	0.08	16.98	3.78E-05	17.20	3.36E-05	1.00	

Marker	Chr	Bp hg19	Al1	A freq1	Or al1	Al2	A freq2	Or al2	Std err	Wald chisq	Pvalue	Lr chisq	Lr pval	R sqr	Gene name
rs3110644	17	36073196	T	0.78	0.67	G	0.22	1.50	0.10	16.95	3.83E-05	16.97	3.80E-05	0.93	*HNF1B*
rs1940245	9	113522917	C	0.72	0.69	A	0.28	1.44	0.09	16.88	3.99E-05	16.93	3.88E-05	0.99	*MUSK*
rs12154794	7	41301890	C	0.68	1.45	G	0.32	0.69	0.09	16.87	3.99E-05	17.33	3.15E-05	0.92	
rs10016656	4	131603386	C	0.80	0.67	T	0.20	1.50	0.10	16.86	4.02E-05	16.83	4.08E-05	0.96	
rs9297402	8	108853863	T	0.92	0.55	C	0.08	1.83	0.15	16.85	4.04E-05	16.81	4.14E-05	0.99	
rs10187793	2	40796854	T	0.97	0.38	C	0.03	2.66	0.24	16.83	4.09E-05	16.84	4.08E-05	0.82	*SLC8A1*
rs4680748	3	28293360	G	0.70	1.45	C	0.30	0.69	0.09	16.83	4.09E-05	17.30	3.20E-05	0.98	*CMC1*
rs17026180	2	40783776	A	0.94	0.48	C	0.06	2.08	0.18	16.83	4.10E-05	16.99	3.75E-05	0.84	*SLC8A1*
rs1338525	1	109368333	G	0.48	0.73	A	0.52	1.38	0.08	16.82	4.11E-05	17.07	3.61E-05	0.98	*AKNAD1*
rs11986704	8	108842979	G	0.92	0.55	T	0.08	1.83	0.15	16.75	4.26E-05	16.70	4.37E-05	0.97	
rs13260753	8	119438828	C	0.69	0.70	A	0.31	1.43	0.09	16.75	4.27E-05	16.82	4.10E-05	0.96	*SAMD12*
rs937295	8	108848708	G	0.92	0.55	A	0.08	1.83	0.15	16.74	4.28E-05	16.69	4.40E-05	0.98	
rs7835295	8	119468609	A	0.70	0.70	G	0.30	1.42	0.09	16.70	4.38E-05	16.77	4.22E-05	1.00	*SAMD12*
rs1016598	6	5159417	G	0.66	1.47	A	0.34	0.68	0.09	16.69	4.40E-05	17.08	3.59E-05	0.86	*LYRM4*
rs1565924	4	11511452	A	0.65	0.71	G	0.35	1.41	0.08	16.67	4.45E-05	16.78	4.21E-05	1.00	
rs4629902	8	119444843	C	0.69	0.70	A	0.31	1.43	0.09	16.63	4.54E-05	16.70	4.37E-05	0.97	*SAMD12*
rs4492409	8	119444880	T	0.69	0.70	C	0.31	1.43	0.09	16.62	4.57E-05	16.69	4.40E-05	0.97	*SAMD12*
rs16867700	5	38754629	C	0.90	0.60	T	0.10	1.68	0.13	16.59	4.65E-05	16.52	4.81E-05	0.98	
rs9425178	1	80673138	C	0.59	0.71	T	0.41	1.42	0.09	16.59	4.65E-05	16.77	4.23E-05	0.96	
rs6122434	20	62014997	G	0.78	1.51	A	0.22	0.66	0.10	16.56	4.70E-05	17.17	3.43E-05	1.00	
rs4876416	8	119446504	T	0.69	0.70	G	0.31	1.43	0.09	16.53	4.79E-05	16.60	4.61E-05	0.97	*SAMD12*
rs169421	13	103747532	C	0.47	1.39	A	0.53	0.72	0.08	16.47	4.94E-05	16.66	4.48E-05	1.00	
rs12024171	1	14567691	T	0.68	1.43	C	0.32	0.70	0.09	16.45	4.99E-05	16.87	4.00E-05	1.00	
rs3110645	17	36073176	T	0.79	0.67	G	0.21	1.50	0.10	16.45	4.99E-05	16.45	4.99E-05	0.95	*HNF1B*
rs2460842	15	36178462	G	0.80	0.67	A	0.20	1.48	0.10	16.44	5.03E-05	16.42	5.07E-05	1.00	
rs6469747	8	119465210	G	0.70	0.70	A	0.30	1.42	0.09	16.43	5.05E-05	16.50	4.88E-05	1.00	*SAMD12*
rs1194743	10	54212597	C	0.72	1.47	T	0.28	0.68	0.09	16.42	5.08E-05	16.86	4.03E-05	0.97	
rs4876845	8	119464447	G	0.70	0.70	A	0.30	1.42	0.09	16.40	5.12E-05	16.47	4.94E-05	1.00	*SAMD12*
rs3918310	13	102609607	C	0.77	0.68	T	0.23	1.47	0.10	16.39	5.16E-05	16.39	5.15E-05	0.96	*FGF14*
rs7810180	7	52516860	G	0.66	1.42	A	0.34	0.70	0.09	16.35	5.27E-05	16.75	4.26E-05	0.99	
rs7667982	4	7936522	A	0.88	0.61	G	0.12	1.63	0.12	16.35	5.27E-05	16.31	5.36E-05	1.00	*AFAP1*
rs10981589	9	115759519	G	0.95	2.46	A	0.05	0.41	0.22	16.32	5.36E-05	18.89	1.39E-05	0.99	*ZNF883*
rs4876840	8	119463071	G	0.70	0.71	A	0.30	1.42	0.09	16.31	5.38E-05	16.37	5.20E-05	1.00	*SAMD12*
rs10491741	9	12799028	C	0.87	0.63	A	0.13	1.60	0.12	16.31	5.39E-05	16.28	5.48E-05	0.99	*LURAP1L*
rs11987403	8	108856341	A	0.92	0.55	G	0.08	1.81	0.15	16.27	5.48E-05	16.22	5.65E-05	0.98	
rs1425118	2	216958899	C	0.75	0.69	G	0.25	1.44	0.09	16.24	5.60E-05	16.31	5.38E-05	0.99	*TMEM169*
rs6982369	8	119470962	G	0.70	0.70	A	0.30	1.43	0.09	16.23	5.61E-05	16.29	5.43E-05	0.94	*SAMD12*
rs7737513	5	136028557	G	0.57	1.39	C	0.43	0.72	0.08	16.19	5.72E-05	16.49	4.89E-05	1.00	
rs1858909	5	136030301	T	0.57	1.39	C	0.43	0.72	0.08	16.18	5.77E-05	16.47	4.94E-05	1.00	
rs4380648	5	136029849	C	0.57	1.39	T	0.43	0.72	0.08	16.18	5.77E-05	16.47	4.95E-05	1.00	
rs13106050	4	11480372	G	0.63	0.71	A	0.37	1.42	0.09	16.14	5.88E-05	16.24	5.58E-05	0.93	

Marker	Chr	Bp hg19	Al1	A freq1	Or al1	Al2	A freq2	Or al2	Std err	Wald chisq	Pvalue	Lr chisq	Lr pval	R sqr	Gene name
rs13251719	8	119438089	T	0.66	0.71	C	0.34	1.42	0.09	16.14	5.89E-05	16.23	5.62E-05	0.93	*SAMD12*
rs2037347	15	78558509	A	0.83	0.66	G	0.17	1.52	0.10	16.13	5.92E-05	16.08	6.08E-05	1.00	*DNAJA4*
rs7049103	9	3824004	G	0.89	1.77	A	0.11	0.57	0.14	16.12	5.94E-05	17.14	3.48E-05	0.97	*GLIS3*
rs1389983	8	108856834	C	0.92	0.55	T	0.08	1.81	0.15	16.12	5.96E-05	16.06	6.15E-05	0.98	
rs2305638	3	47046842	C	0.51	1.39	G	0.49	0.72	0.08	16.08	6.08E-05	16.30	5.40E-05	0.97	*NBEAL2*
rs1733724	10	54223977	G	0.72	1.45	A	0.28	0.69	0.09	16.08	6.09E-05	16.49	4.90E-05	0.99	
rs1866658	2	127443627	G	0.68	1.45	A	0.33	0.69	0.09	16.07	6.10E-05	16.47	4.95E-05	0.88	*GYPC*
rs4737478	8	58758894	C	0.82	0.64	T	0.18	1.56	0.11	16.06	6.13E-05	16.06	6.14E-05	0.86	
rs13098603	3	47534109	G	0.67	0.69	A	0.33	1.46	0.09	16.06	6.15E-05	16.18	5.77E-05	0.81	
rs4297099	9	113476105	C	0.68	0.72	T	0.32	1.39	0.08	16.05	6.16E-05	16.12	5.96E-05	1.00	*MUSK*
rs11666877	19	46777507	C	0.68	0.69	G	0.32	1.45	0.09	16.04	6.20E-05	16.11	5.98E-05	0.88	
rs173549	16	84543067	C	0.97	0.41	T	0.03	2.44	0.22	16.04	6.20E-05	16.42	5.08E-05	0.95	
rs247856	16	84542415	G	0.97	0.40	T	0.03	2.48	0.23	16.03	6.24E-05	16.42	5.08E-05	0.93	
rs7348332	20	4640900	A	0.98	0.33	G	0.02	2.99	0.27	16.01	6.29E-05	16.49	4.89E-05	0.97	
rs4782986	16	84543409	C	0.97	0.41	T	0.03	2.42	0.22	16.01	6.30E-05	16.38	5.18E-05	0.96	
rs7495580	15	27466391	A	0.89	0.61	G	0.11	1.65	0.13	16.00	6.33E-05	15.98	6.41E-05	1.00	*GABRG3*
rs4782997	16	84553410	T	0.97	0.42	A	0.03	2.37	0.22	15.96	6.46E-05	16.34	5.30E-05	0.99	
rs17784127	3	47061700	T	0.55	1.38	C	0.45	0.72	0.08	15.96	6.47E-05	16.20	5.69E-05	1.00	*SETD2*
rs7610636	3	47064436	A	0.52	1.38	C	0.48	0.73	0.08	15.96	6.47E-05	16.19	5.74E-05	1.00	*SETD2*
rs1545195	7	41307633	T	0.70	1.42	A	0.30	0.70	0.09	15.96	6.49E-05	16.38	5.19E-05	0.99	
rs4782990	16	84544538	A	0.97	0.42	G	0.03	2.39	0.22	15.95	6.49E-05	16.32	5.36E-05	0.97	
rs4782991	16	84544818	G	0.97	0.42	A	0.03	2.38	0.22	15.95	6.51E-05	16.31	5.37E-05	0.97	
rs2385867	3	47059323	T	0.52	1.38	C	0.48	0.72	0.08	15.94	6.53E-05	16.17	5.80E-05	1.00	*SETD2*
rs11920354	3	47262246	C	0.56	1.38	A	0.44	0.73	0.08	15.94	6.54E-05	16.19	5.72E-05	1.00	
rs11768187	7	41304710	C	0.70	1.42	T	0.30	0.70	0.09	15.92	6.60E-05	16.34	5.28E-05	0.99	
rs4782998	16	84553507	G	0.97	0.42	T	0.03	2.36	0.22	15.92	6.60E-05	16.30	5.42E-05	0.99	
rs7632501	3	47268306	T	0.56	1.38	C	0.44	0.73	0.08	15.92	6.62E-05	16.17	5.79E-05	1.00	*KIF9*
rs11715121	3	47072621	T	0.55	1.38	A	0.45	0.73	0.08	15.89	6.73E-05	16.13	5.93E-05	1.00	*SETD2*
rs4782999	16	84553537	G	0.97	0.42	C	0.03	2.36	0.22	15.88	6.75E-05	16.25	5.54E-05	0.99	
rs6768221	3	47296899	C	0.56	1.38	G	0.44	0.73	0.08	15.87	6.78E-05	16.12	5.93E-05	0.99	*KIF9*
rs7611348	3	2921102	A	0.65	0.72	G	0.35	1.40	0.08	15.87	6.78E-05	15.95	6.52E-05	0.98	*CNTN4*
rs12672313	7	115956430	C	0.87	1.66	A	0.13	0.60	0.13	15.86	6.82E-05	16.84	4.07E-05	0.99	
rs11130126	3	47306894	A	0.56	1.38	G	0.44	0.73	0.08	15.86	6.82E-05	16.11	5.97E-05	0.99	*KIF9*
rs10955877	8	119436884	A	0.71	0.70	G	0.29	1.43	0.09	15.86	6.82E-05	15.92	6.62E-05	0.95	*SAMD12*
rs952133	1	56859419	C	0.94	0.52	A	0.06	1.94	0.17	15.86	6.83E-05	15.69	7.47E-05	1.00	
rs4397486	9	113478462	G	0.68	0.72	T	0.32	1.39	0.08	15.84	6.88E-05	15.90	6.67E-05	1.00	*MUSK*
rs17138596	7	115955097	T	0.87	1.66	C	0.13	0.60	0.13	15.84	6.89E-05	16.82	4.12E-05	0.98	
rs7826257	8	119461395	G	0.70	0.71	A	0.30	1.41	0.09	15.83	6.92E-05	15.89	6.71E-05	1.00	*SAMD12*
rs11920853	3	47308183	G	0.56	1.38	A	0.44	0.73	0.08	15.82	6.95E-05	16.07	6.09E-05	0.99	*KIF9*
rs16974016	16	84549970	G	0.97	0.43	A	0.03	2.34	0.21	15.82	6.95E-05	16.18	5.77E-05	1.00	
rs10266118	7	52520577	T	0.69	1.44	C	0.31	0.70	0.09	15.82	6.97E-05	16.24	5.59E-05	0.93	

Marker	Chr	Bp hg19	Al1	A freq1	Or al1	Al2	A freq2	Or al2	Std err	Wald chisq	Pvalue	Lr chisq	Lr pval	R sqr	Gene name
rs4864014	4	131599180	A	0.80	0.68	G	0.21	1.47	0.10	15.81	6.99E-05	15.78	7.11E-05	1.00	
rs7825963	8	119461193	C	0.70	0.71	A	0.30	1.41	0.09	15.81	7.00E-05	15.87	6.79E-05	1.00	*SAMD12*
rs4863834	4	131599136	G	0.79	0.68	T	0.21	1.47	0.10	15.80	7.02E-05	15.77	7.15E-05	1.00	
rs11915074	3	47309757	A	0.56	1.38	T	0.44	0.73	0.08	15.80	7.04E-05	16.05	6.17E-05	0.99	*KIF9*
rs10092441	8	108858606	T	0.92	0.56	C	0.08	1.80	0.15	15.79	7.08E-05	15.72	7.33E-05	0.98	
rs10955886	8	119459334	C	0.70	0.71	G	0.30	1.41	0.09	15.77	7.15E-05	15.83	6.94E-05	1.00	*SAMD12*
rs11972531	7	52519258	A	0.69	1.43	G	0.31	0.70	0.09	15.77	7.16E-05	16.18	5.76E-05	0.95	
rs6721207	2	216958412	G	0.75	0.70	A	0.25	1.43	0.09	15.76	7.17E-05	15.83	6.93E-05	0.99	*TMEM169*
rs17053372	8	25163078	C	0.95	0.47	T	0.05	2.11	0.19	15.74	7.28E-05	15.66	7.58E-05	0.83	*DOCK5*
rs9845728	3	47103376	T	0.55	1.38	C	0.45	0.73	0.08	15.73	7.32E-05	15.96	6.46E-05	1.00	*SETD2*
rs1004811	22	33809429	C	0.62	1.40	T	0.38	0.72	0.09	15.72	7.35E-05	16.01	6.30E-05	1.00	*LARGE*
rs247858	16	84540654	C	0.97	0.39	A	0.03	2.54	0.24	15.72	7.36E-05	16.11	5.98E-05	0.92	
rs9844122	3	47108330	T	0.55	1.38	C	0.45	0.73	0.08	15.71	7.37E-05	15.95	6.50E-05	1.00	*SETD2*
rs577938	16	84553973	A	0.97	0.42	G	0.03	2.36	0.22	15.71	7.37E-05	16.08	6.08E-05	0.99	
rs12155345	7	41301734	A	0.70	1.42	C	0.30	0.71	0.09	15.71	7.40E-05	16.12	5.96E-05	1.00	
rs10108907	8	119454157	G	0.70	0.71	A	0.30	1.41	0.09	15.70	7.41E-05	15.76	7.19E-05	1.00	*SAMD12*
rs10488329	7	52528988	C	0.69	1.42	A	0.31	0.70	0.09	15.70	7.44E-05	16.10	6.02E-05	0.97	
rs10955878	8	119440005	T	0.67	0.71	C	0.33	1.40	0.09	15.64	7.64E-05	15.72	7.33E-05	0.96	*SAMD12*
rs1528243	7	115970664	G	0.87	1.65	A	0.13	0.61	0.13	15.64	7.68E-05	16.58	4.66E-05	1.00	
rs12540030	7	115976844	A	0.87	1.65	C	0.13	0.61	0.13	15.63	7.70E-05	16.58	4.67E-05	0.99	
rs6606889	15	27467814	G	0.89	0.61	A	0.11	1.64	0.13	15.63	7.70E-05	15.60	7.82E-05	0.99	*GABRG3*
rs1205322	19	54844437	G	0.44	0.72	A	0.56	1.39	0.08	15.63	7.70E-05	15.88	6.74E-05	1.00	*LILRA4*
rs10955879	8	119440018	G	0.67	0.71	C	0.33	1.40	0.09	15.62	7.73E-05	15.70	7.42E-05	0.96	*SAMD12*
rs10112933	8	108859944	G	0.92	0.56	A	0.08	1.80	0.15	15.62	7.75E-05	15.55	8.02E-05	0.98	
rs11773916	8	119440931	C	0.67	0.71	T	0.33	1.40	0.09	15.61	7.78E-05	15.69	7.47E-05	0.96	*SAMD12*
rs12590361	14	103202717	C	0.92	1.86	T	0.08	0.54	0.16	15.61	7.80E-05	17.01	3.73E-05	1.00	
rs12156430	8	119442897	C	0.67	0.71	A	0.33	1.40	0.09	15.59	7.85E-05	15.67	7.54E-05	0.96	*SAMD12*
rs2072569	2	40783227	A	0.90	0.57	T	0.10	1.76	0.14	15.59	7.86E-05	15.54	8.09E-05	0.82	*SLC8A1*
rs7167162	15	36182613	A	0.80	0.68	G	0.20	1.47	0.10	15.59	7.88E-05	15.56	7.99E-05	1.00	
rs4748314	10	16767367	T	0.45	1.37	C	0.55	0.73	0.08	15.58	7.90E-05	15.74	7.27E-05	1.00	*RSU1*
rs7167757	15	36182930	A	0.80	0.68	T	0.20	1.47	0.10	15.56	7.98E-05	15.54	8.09E-05	1.00	
rs4740532	9	12816938	G	0.83	0.66	C	0.17	1.51	0.10	15.56	7.98E-05	15.54	8.10E-05	0.99	*LURAP1L*
rs4548208	8	119443712	G	0.67	0.71	A	0.33	1.40	0.09	15.56	7.99E-05	15.64	7.67E-05	0.96	*SAMD12*
rs10480976	7	52520099	T	0.70	1.43	G	0.30	0.70	0.09	15.55	8.04E-05	15.96	6.48E-05	0.95	
rs923997	4	11500166	A	0.66	0.71	G	0.34	1.40	0.09	15.55	8.05E-05	15.63	7.70E-05	1.00	
rs7838464	8	119444368	C	0.67	0.71	G	0.33	1.40	0.09	15.54	8.06E-05	15.62	7.74E-05	0.96	*SAMD12*
rs17114029	1	56961955	T	0.96	0.44	C	0.04	2.26	0.21	15.54	8.09E-05	15.66	7.58E-05	0.95	*PPAP2B*
rs4916638	4	11496628	A	0.66	0.71	T	0.34	1.40	0.09	15.53	8.14E-05	15.61	7.79E-05	1.00	
rs6842997	4	11493565	G	0.66	0.71	C	0.34	1.40	0.09	15.51	8.19E-05	15.60	7.85E-05	1.00	
rs4631494	8	119445009	G	0.67	0.71	C	0.33	1.40	0.09	15.50	8.27E-05	15.57	7.94E-05	0.97	*SAMD12*
rs4523590	10	16748793	C	0.46	1.38	G	0.54	0.73	0.08	15.49	8.29E-05	15.66	7.59E-05	0.98	*RSU1*

Marker	Chr	Bp hg19	Al1	A freq1	Or al1	Al2	A freq2	Or al2	Std err	Wald chisq	Pvalue	Lr chisq	Lr pval	R sqr	Gene name
rs6785790	3	47146932	T	0.52	1.37	C	0.48	0.73	0.08	15.48	8.33E-05	15.69	7.45E-05	1.00	*SETD2*
rs7012582	8	119457524	T	0.68	0.71	A	0.32	1.40	0.09	15.47	8.38E-05	15.54	8.08E-05	0.98	*SAMD12*
rs9642841	8	119445360	T	0.67	0.72	C	0.33	1.40	0.09	15.47	8.40E-05	15.54	8.08E-05	0.97	*SAMD12*
rs4443692	8	119449083	T	0.70	0.71	C	0.30	1.41	0.09	15.46	8.43E-05	15.51	8.19E-05	0.99	*SAMD12*
rs9643130	8	119445380	C	0.67	0.72	T	0.33	1.40	0.09	15.45	8.47E-05	15.53	8.14E-05	0.97	*SAMD12*
rs10103556	8	108860895	T	0.92	0.56	C	0.08	1.79	0.15	15.45	8.49E-05	15.38	8.80E-05	0.97	
rs2132151	4	11487955	G	0.66	0.71	T	0.34	1.40	0.09	15.44	8.50E-05	15.52	8.15E-05	0.99	
rs7813395	8	119446196	A	0.67	0.72	G	0.33	1.40	0.09	15.44	8.53E-05	15.51	8.20E-05	0.97	*SAMD12*
rs6749415	2	216958402	A	0.75	0.70	G	0.25	1.43	0.09	15.43	8.56E-05	15.49	8.29E-05	0.99	*TMEM169*
rs4789807	17	80556103	C	0.82	1.59	T	0.18	0.63	0.12	15.42	8.60E-05	16.05	6.17E-05	0.83	*FOXK2*
rs2276854	3	47276968	T	0.53	1.37	C	0.47	0.73	0.08	15.42	8.60E-05	15.64	7.66E-05	1.00	*KIF9*
rs7644112	3	2921135	C	0.66	0.72	A	0.34	1.39	0.08	15.41	8.63E-05	15.48	8.35E-05	0.99	*CNTN4*
rs6442762	3	2921461	G	0.66	0.72	T	0.34	1.39	0.09	15.41	8.65E-05	15.47	8.36E-05	0.96	*CNTN4*
rs1547471	7	52517417	G	0.69	1.41	A	0.31	0.71	0.09	15.41	8.65E-05	15.80	7.03E-05	1.00	
rs13094438	3	47277885	A	0.53	1.37	G	0.47	0.73	0.08	15.41	8.66E-05	15.63	7.72E-05	1.00	*KIF9*
rs431551	16	84538472	T	0.97	0.40	G	0.03	2.50	0.23	15.40	8.68E-05	15.84	6.91E-05	0.84	*TLDC1*
rs17114017	1	56960360	G	0.96	0.45	A	0.04	2.21	0.20	15.40	8.69E-05	15.55	8.05E-05	0.98	*PPAP2B*
rs13255144	8	119447015	C	0.67	0.72	T	0.33	1.40	0.09	15.40	8.70E-05	15.47	8.36E-05	0.97	*SAMD12*
rs6791842	3	47279810	C	0.53	1.37	T	0.47	0.73	0.08	15.40	8.72E-05	15.61	7.77E-05	1.00	*KIF9*
rs10894626	11	132815368	G	0.57	0.72	A	0.43	1.39	0.08	15.39	8.73E-05	15.54	8.08E-05	0.99	*OPCML*
rs8026989	15	27469198	C	0.89	0.61	G	0.11	1.64	0.13	15.39	8.74E-05	15.36	8.90E-05	0.99	*GABRG3*
rs10283133	8	119447420	G	0.67	0.72	A	0.33	1.40	0.09	15.38	8.79E-05	15.45	8.46E-05	0.97	*SAMD12*
rs11130123	3	47285912	G	0.53	1.37	A	0.47	0.73	0.08	15.37	8.83E-05	15.59	7.87E-05	1.00	*KIF9*
rs2276853	3	47282303	A	0.53	1.37	G	0.47	0.73	0.08	15.37	8.85E-05	15.58	7.89E-05	1.00	*KIF9*
rs4639131	4	68095870	C	0.57	1.38	T	0.43	0.72	0.08	15.35	8.93E-05	15.60	7.82E-05	1.00	
rs11130124	3	47292183	C	0.53	1.37	T	0.47	0.73	0.08	15.35	8.94E-05	15.57	7.97E-05	0.99	*KIF9*
rs8141754	22	33810205	T	0.62	1.39	A	0.38	0.72	0.09	15.34	9.00E-05	15.61	7.77E-05	1.00	*LARGE*
rs4082155	3	47125385	A	0.52	1.37	G	0.48	0.73	0.08	15.33	9.03E-05	15.54	8.09E-05	1.00	*SETD2*
rs9310864	3	28450371	C	0.66	1.39	T	0.34	0.72	0.09	15.32	9.06E-05	15.66	7.59E-05	1.00	*ZCWPW2*
rs7012122	8	62330609	T	0.93	0.55	C	0.07	1.81	0.15	15.31	9.12E-05	15.31	9.13E-05	0.99	*CLVS1*
rs4078466	3	47126543	T	0.52	1.37	C	0.48	0.73	0.08	15.31	9.14E-05	15.51	8.19E-05	1.00	*SETD2*
rs8138344	22	33820256	G	0.62	1.39	T	0.38	0.72	0.08	15.29	9.20E-05	15.57	7.94E-05	1.00	*LARGE*
rs4683326	3	47292827	A	0.54	1.38	G	0.46	0.73	0.08	15.22	9.55E-05	15.44	8.52E-05	0.96	*KIF9*
rs2986575	1	183907670	G	0.73	0.71	A	0.27	1.41	0.09	15.22	9.56E-05	15.25	9.41E-05	0.99	*COLGALT2*
rs6702829	1	29766241	T	0.77	0.70	C	0.23	1.43	0.09	15.20	9.69E-05	15.18	9.75E-05	0.98	
rs753960	17	3831240	C	0.84	1.60	T	0.16	0.63	0.12	15.17	9.82E-05	15.92	6.60E-05	0.90	*ATP2A3*

To retrieve information about SNPs and their genomic context (the nearest gene and location) we used the hg18 (NCBI 36) assembly. We performed a logistic regression under an additive model on SNP dosage levels adjusting for sex and the first four principal components, using mach2dat software. The SNPs are ranked for p-value.

S2 Table. **eQTL analysis chr 2**.

Gene Symbol	marker	Rs id	CRBL	FCTX	HIPP	MEDU	OCTX	PUTM	SNIG	TCTX	THAL	WHMT
LOC730100	chr2:52532874	rs12619513	3.60E-01	1.40E-01	2.70E-02	3.20E-03	9.90E-01	7.50E-01	8.60E-01	9.90E-02	8.50E-01	3.90E-01
LOC730100	chr2:52532874	rs12619513	8.20E-01	8.10E-01	9.30E-01	3.40E-01	8.50E-01	6.90E-02	3.40E-01	5.90E-02	2.30E-01	8.20E-01
LOC730100	chr2:52532874	rs12619513	7.00E-01	6.80E-02	1.50E-01	2.20E-01	9.60E-01	1.70E-01	8.90E-01	3.50E-01	7.90E-01	1.40E-01
LOC730100	chr2:52532874	rs12619513	9.00E-01	8.70E-01	5.70E-01	8.80E-02	9.30E-01	6.40E-01	8.60E-01	8.00E-02	7.80E-01	7.80E-01
LOC730100	chr2:52532874	rs12619513	2.80E-01	5.20E-01	7.10E-01	1.60E-01	1.00E+00	8.90E-02	5.80E-01	9.60E-02	9.30E-01	7.50E-01
LOC730100	chr2:52532874	rs12619513	6.30E-01	9.90E-01	5.80E-01	7.30E-01	9.60E-01	6.90E-01	9.30E-02	5.40E-01	2.20E-01	7.20E-01
LOC730100	chr2:52532874	rs12619513	7.40E-01	2.50E-01	9.20E-01	1.20E-01	5.30E-01	5.30E-01	1.50E-01	2.10E-01	2.90E-01	8.70E-01
LOC730100	chr2:52532874	rs12619513	1.50E-01	7.80E-01	3.50E-01	1.80E-01	9.20E-01	9.00E-01	5.40E-01	2.30E-01	8.20E-01	9.30E-01
LOC730100	chr2:52532874	rs12619513	1.00E+00	5.10E-01	8.40E-01	4.00E-01	5.30E-01	8.30E-01	1.60E-01	8.40E-01	8.30E-01	3.00E-01

Summary of the eQTL exerted by rs12619513 in 10 different assayed brain tissues. CRBL = cerebellum; FCTX = frontal cortex; HIPP = hippocampus; MEDU = medulla; OCTX = occipital cortex; PUTM = putamen; SNIG = substantia nigra; TCTX = temporal cortex; THAL = thalamus; WHMT = white matter.

S3 Table. **eQTL analysis chr 17**.

Marker	Chr	BP	Risk allele	*Cis* effect	Target gene	Brain region	p-value
rs906175	17	79173462	T	decreased expression	*RFNG*	hippocampus	5.60E-06
					AATK<br>*MIR1250*	temporal cortex	6.30E-05
rs2725391		79192430	T	decreased expression	*RFNG*	hippocampus	9.50E-05
					AATK<br>*MIR1250*	temporal cortex	6.30E-05
rs969413		79195814	A	decreased expression	*RFNG*	hippocampus	8.20E-05
					AATK<br>*MIR1250*	temporal cortex	9.30E-05
rs2659030		79177974	A	decreased expression	*RFNG*	hippocampus	7.70E-06
					AATK<br>*MIR1250*	temporal cortex	1.50E-04
rs2255166		79213562	C	decreased expression	*RFNG*	hippocampus	6.40E-05
rs9319617		79192446	T	decreased expression	*RFNG*	hippocampus	7.10E-04
					AATK<br>*MIR1250*	temporal cortex	2.70E-05
rs1048775		79202329	C	decreased expression	*AATK*<br>*MIR1250*	temporal cortex	5.10E-04

Summary of the eQTL exerted by rs906175, rs2725391, rs969413, rs2659030, rs2255166, rs9319617 and rs1048775. The risk allele of each SNP exerts a significant (p-value < 10^{-5}) or suggestive (p-value > 10^{-5}) on cis target genes such as *RFNG*, *AATK* and *MIR1250*.

S4 Table. **List of disease associated genes (DAGs)**.

Gene	EntrezID
A2BP1	54715
ACSBG1	23205
ACTC1	70
ADCK5	203054
AFAP1	60312
AGK	55750
AK5	26289
ARHGAP10	79658
ARHGAP15	55843
ARRB2	409
ASGR2	433
ATP1A2	477
ATP2A3	489
ATP2C2	9914
ATRNL1	26033
CEP131	22994
B3GAT2	135152
BCAS4	55653
BCL2L14	79370
BNC2	54796
BOK	666
BPIL2	254240
C12orf40	283461
C15orf27	123591
ENTHD2	146705
C17orf89	284184
C1orf21	81563
C1orf62	254268
C1QTNF7	114905
C2orf56	55471
C5orf20	140947
C9orf126	286205
C9orf150	286343
C9orf3	84909
C9orf57	138240
CADPS	8618
CAMTA1	23261
CCDC12	151903
CCDC6	8030

Gene	EntrezID
CCDC81	60494
CCDC91	55297
CCNY	219771
CD96	10225
CDH20	28316
CDKN3	1033
CEBPZ	10153
CENPC1	1060
CHCHD6	84303
CHL1	10752
CIB2	10518
CLASP1	23332
CLCN2	1181
CMC1	152100
CNOT2	4848
CNTN4	152330
CNTN5	53942
COL24A1	255631
COL9A1	1297
CPNE3	8895
CPSF1	29894
CSMD1	64478
DCHS2	54798
DGKI	9162
DHRS3	9249
DLL1	28514
DNAH7	56171
DNAJA4	55466
DOCK2	1794
DSCAML1	57453
DUSP15	128853
DYSF	8291
ELK3	2004
ENPP1	5167
ERC2	26059
EXPH5	23086
FAM26D	221301
FAM92B	339145
FARP2	9855
FBXL6	26233
FBXO25	26260
FGF11	2256

Gene	EntrezID
FGF14	2259
FOXK2	3607
FREM2	341640
GABRG3	2567
GALM	130589
GATA4	2626
GLT25D2	23127
GLTSCR1	29998
GMPR	2766
GP2	2813
GPC5	2262
GPR172A	79581
GRID1	2894
GRIN2B	2904
GRM6	2916
GYPC	2995
HEG1	57493
HNF1B	6928
HOMER2	9455
HS6ST3	266722
IDH3A	3419
IGSF21	84966
IL1R1	3554
IL1RL1	9173
INADL	10207
INPP4B	8821
IRF8	3394
ITGA1	3672
ITPR1	3708
KCNK4	50801
KCNMA1	3778
KCNMB4	27345
KIAA0090	23065
KIAA0247	9766
KIAA0746	23231
KIAA1026	23254
KIAA1486	57624
KIF19	124602
KIF9	64147
KLHL18	23276
KRT20	54474
KRT26	353288

Gene	EntrezID
LARGE	9215
LBR	3930
LDLRAD3	143458
LIFR	3977
LILRA4	23547
LOC388419	388419
LOC642502	642502
LOC646892	646892
LPAR3	23566
LPP	4026
LRP1	4035
LRP1B	53353
LYRM4	57128
MAML2	84441
MAP1B	4131
MAPK8	5599
MBD5	55777
MDGA2	161357
MEIS1	4211
MKI67IP	84365
MUSK	4593
MX1	4599
MYO10	4651
NAP5	344148
NAV1	89796
NBEAL2	23218
NCAM2	4685
NCOA7	135112
NEIL2	252969
NFAM1	150372
NHLH2	4808
NLGN2	57555
NMNAT3	349565
NRXN3	9369
NUDT7	283927
NUP50	10762
OAS2	4939
OPCML	4978
OR10G3	26533
ORC5L	5001
PADI1	29943
PAX5	5079

Gene	EntrezID
PCDH15	65217
PCSK2	5126
PCSK5	5125
PCYT1A	5130
PDE10A	10846
PDE8A	5151
PDRG1	81572
PEBP4	157310
PECR	55825
PELI2	57161
PGLYRP2	114770
PHF21B	112885
PIB5PA	27124
PLA2G3	50487
PLCB1	23236
PLCB3	5331
PLOD3	8985
PMFBP1	83449
PNPLA5	150379
PON1	5444
POU5F1	5460
PPA2	27068
PRKD3	23683
PRKG1	5592
PRND	23627
PSG2	5670
PTGER3	5733
PTHR1	5745
PTPN23	25930
PTPRG	5793
PVRL2	5819
QPCT	25797
RAB28	9364
RAB30	27314
RAB40B	10966
RASA3	22821
RASD2	23551
RASGRF1	5923
RCBTB1	55213
RELN	5649
REV3L	5980
RFX3	5991

Gene	EntrezID
RGS7	6000
ROR2	4920
RPL30	6156
RPS6KA2	6196
RSU1	6251
RXRA	6256
SAMD12	401474
SCAP	22937
SCGB1D4	404552
SDHC	6391
SETD2	29072
SGCG	6445
SIAH2	6478
SLC19A2	10560
SLC38A10	124565
SLC39A10	57181
SLC7A13	157724
SLIT3	6586
SP100	6672
SPHKAP	80309
SPOCK3	50859
ST3GAL1	6482
SULT6B1	391365
SYCP2	10388
SYNGR1	9145
TCERG1L	256536
TCF7L2	6934
TCL6	27004
TIFAB	497189
TM9SF2	9375
TMEM103	54859
TMEM169	92691
TMEM63C	57156
TMOD1	7111
TMTC3	160418
TNFRSF11A	8792
TOMM40	10452
TSPAN14	81619
TTC23L	153657
TTC39B	158219
TTC7A	57217
TTLL9	164395

Gene	EntrezID
TUBA1C	84790
TUSC3	7991
UBE2E1	7324
UBE2G1	7326
UGP2	7360
ULBP1	80329
VAV2	7410
VCAM1	7412
VN1R2	317701
VN1R4	317703
VPS54	51542
WDR21B	285429
WDR45L	56270
WDR61	80349
WNT3	7473
XKR8	55113
XRCC5	7520
ZCCHC24	219654
ZCWPW2	152098
ZHX2	22882
ZNF765	91661
ZNF835	90485
CLVS1	157807
DOCK5	80005
GLIS3	169792
LOC730100	730100
PPAP2B	8613
SLC8A1	6546
TLDC1	57707
TMEM184C	55751
ZNF883	169834

Gene list (271 from genes-based and 9 non overlapping genes from SNPs-based analysis) with HugoID and EntrezID with p-value < 10^{-1}, after FDR correction with the Benjamini & Hochberg procedure.

S5 Table. Functional annotation cluster analysis.

Annotation Cluster 1	Enrichment Score: 2.09, average p-value=0.00813						
**Category**	**Term**	**Count**	**%**	**p-value**	**% List**	**% Genome**	**Fold Enrichment**
GOTERM_BP_ALL	GO:0048731~system development	54	19.35483871	2.76E-04	26.60098522	16.50609238	1.611585869
GOTERM_BP_ALL	GO:0048856~anatomical structure development	57	20.43010753	3.44E-04	28.07881773	17.90167186	1.568502537
GOTERM_BP_ALL	GO:0048468~cell development	21	7.52688172	9.41E-04	10.34482759	4.562198923	2.267509103
GOTERM_BP_ALL	GO:0031175~neuron projection development	12	4.301075269	0.001162356	5.911330049	1.813544914	3.259544335
GOTERM_BP_ALL	GO:0048666~neuron development	14	5.017921147	0.00120934	6.896551724	2.401530179	2.871732275
GOTERM_BP_ALL	GO:0032502~developmental process	65	23.29749104	0.001223731	32.01970443	22.30093511	1.435800978
GOTERM_BP_ALL	GO:0007275~multicellular organismal development	60	21.50537634	0.001513732	29.55665025	20.29611788	1.456271116
GOTERM_BP_ALL	GO:0030182~neuron differentiation	16	5.734767025	0.001608319	7.881773399	3.102862001	2.540162404
GOTERM_BP_ALL	GO:0007399~nervous system development	29	10.39426523	0.001738258	14.28571429	7.707565883	1.853466387
GOTERM_BP_ALL	GO:0030030~cell projection organization	14	5.017921147	0.002495616	6.896551724	2.606970813	2.645427286
GOTERM_BP_ALL	GO:0007155~cell adhesion	21	7.52688172	0.002529166	10.34482759	4.958911873	2.086108374
GOTERM_BP_ALL	GO:0022610~biological adhesion	21	7.52688172	0.002570961	10.34482759	4.965996033	2.083132471
GOTERM_BP_ALL	GO:0048699~generation of neurons	18	6.451612903	0.00281753	8.866995074	3.960045339	2.239114534
GOTERM_BP_ALL	GO:0030154~cell differentiation	38	13.62007168	0.003150974	18.71921182	11.59676962	1.614174674
GOTERM_BP_ALL	GO:0048869~cellular developmental process	39	13.97849462	0.003540702	19.21182266	12.08557665	1.58964882
GOTERM_BP_ALL	GO:0048812~neuron projection morphogenesis	10	3.584229391	0.00360067	4.926108374	1.508926041	3.264645343
GOTERM_CC_ALL	GO:0043005~neuron projection	13	4.659498208	0.004666945	5.555555556	2.149861705	2.584145549
GOTERM_BP_ALL	GO:0032989~cellular component morphogenesis	14	5.017921147	0.004757993	6.896551724	2.812411448	2.452184487
GOTERM_BP_ALL	GO:0022008~neurogenesis	18	6.451612903	0.005808487	8.866995074	4.257580051	2.082637312
GOTERM_BP_ALL	GO:0016337~cell-cell adhesion	11	3.94265233	0.006476697	5.418719212	1.95522811	2.771400014
GOTERM_BP_ALL	GO:0009653~anatomical structure morphogenesis	29	10.39426523	0.006605968	14.28571429	8.479739303	1.68468791
GOTERM_BP_ALL	GO:0048858~cell projection morphogenesis	10	3.584229391	0.008798637	4.926108374	1.735619156	2.838242686
GOTERM_BP_ALL	GO:0032990~cell part morphogenesis	10	3.584229391	0.011512067	4.926108374	1.813544914	2.716286946
GOTERM_BP_ALL	GO:0000902~cell morphogenesis	12	4.301075269	0.013591973	5.911330049	2.521960895	2.343941994
GOTERM_BP_ALL	GO:0048513~organ development	37	13.26164875	0.013917191	18.22660099	12.31226976	1.480360757
GOTERM_CC_ALL	GO:0042995~cell projection	19	6.810035842	0.014306661	8.11965812	4.381443299	1.853192559

GOTERM_BP_ALL	GO:0007409~axonogenesis	8	2.867383513	0.021142241	3.9408867	1.367242845	2.882360448
GOTERM_BP_ALL	GO:0032501~multicellular organismal process	76	27.24014337	0.02188572	37.43842365	30.32020402	1.234768197
GOTERM_BP_ALL	GO:0048667~cell morphogenesis involved in neuron differentiation	8	2.867383513	0.030965423	3.9408867	1.480589402	2.661701275
GOTERM_BP_ALL	GO:0007156~homophilic cell adhesion	6	2.150537634	0.040068815	2.955665025	0.928024936	3.184898282
GOTERM_BP_ALL	GO:0006928~cell motion	13	4.659498208	0.04069385	6.403940887	3.364975914	1.903116412
GOTERM_BP_ALL	GO:0000904~cell morphogenesis involved in differentiation	8	2.867383513	0.061957633	3.9408867	1.728534996	2.279899863
GOTERM_BP_ALL	GO:0007411~axon guidance	4	1.433691756	0.19768168	1.97044335	0.758005101	2.599511993
GOTERM_CC_ALL	GO:0030424~axon	5	1.792114695	0.204747025	2.136752137	0.999497108	2.137827232
GOTERM_BP_ALL	GO:0016043~cellular component organization	41	14.6953405	0.239786845	20.19704433	17.69623123	1.141318967
GOTERM_BP_ALL	GO:0007417~central nervous system development	9	3.225806452	0.264031432	4.433497537	3.010767923	1.472547088
GOTERM_BP_ALL	GO:0007420~brain development	5	1.792114695	0.596308971	2.463054187	2.047322188	1.203061346

**Annotation Cluster 2**	**Enrichment Score: 1.83, average p-value=0.01479**						
**Category**	**Term**	**Count**	**%**	**PValue**	**% List**	**% Genome**	**Fold Enrichment**
GOTERM_CC_ALL	GO:0016020~membrane	132	47.31182796	7.28E-04	56.41025641	45.67513201	1.235032148
GOTERM_CC_ALL	GO:0044459~plasma membrane part	47	16.84587814	0.007600139	20.08547009	13.84837817	1.450384286
GOTERM_CC_ALL	GO:0044425~membrane part	116	41.57706093	0.007784709	49.57264957	41.35026402	1.198847232
GOTERM_CC_ALL	GO:0031224~intrinsic to membrane	98	35.12544803	0.013203113	41.88034188	34.47950717	1.214644446
GOTERM_CC_ALL	GO:0005886~plasma membrane	70	25.08960573	0.022358928	29.91452991	23.74277093	1.259942658
GOTERM_CC_ALL	GO:0016021~integral to membrane	89	31.89964158	0.083430466	38.03418803	33.29771184	1.142246296
GOTERM_MF_ALL	GO:0004872~receptor activity	35	12.54480287	0.134643794	15.0862069	12.13762134	1.24292944

**Annotation Cluster 3**	**Enrichment Score: 1.69, average p-value=0.02042**						
**Category**	**Term**	**Count**	**%**	**PValue**	**% List**	**% Genome**	**Fold Enrichment**
GOTERM_CC_ALL	GO:0045202~synapse	14	5.017921147	0.002275887	5.982905983	2.231581594	2.681016011
GOTERM_CC_ALL	GO:0043005~neuron projection	13	4.659498208	0.004666945	5.555555556	2.149861705	2.584145549
GOTERM_CC_ALL	GO:0044456~synapse part	10	3.584229391	0.010507684	4.273504274	1.546391753	2.763532764
GOTERM_BP_ALL	GO:0048167~regulation of synaptic plasticity	5	1.792114695	0.013258561	2.463054187	0.453386228	5.432573892
GOTERM_BP_ALL	GO:0050804~regulation of synaptic transmission	7	2.508960573	0.013502861	3.448275862	0.963445735	3.579107505
GOTERM_BP_ALL	GO:0051969~regulation of transmission of nerve	7	2.508960573	0.019115802	3.448275862	1.041371493	3.311283134

	impulse						
GOTERM_BP_ALL	GO:0031644~regulation of neurological system process	7	2.508960573	0.022766028	3.448275862	1.083876452	3.181428893
GOTERM_BP_ALL	GO:0044057~regulation of system process	10	3.584229391	0.03389372	4.926108374	2.189005384	2.250386596
GOTERM_BP_ALL	GO:0010646~regulation of cell communication	21	7.52688172	0.10669323	10.34482759	7.353357892	1.406816823
GOTERM_BP_ALL	GO:0051239~regulation of multicellular organismal process	16	5.734767025	0.364412907	7.881773399	6.63785775	1.187397154

DAVID Functional annotation clusters with enrichment score (ES) > 1.3 (ES = -log10 (geometric mean (p-values)). Legend: Count = Number of the disease associated genes (DAGs) in the GO term. % = Percent of DAGs in the GO term; Pvalue = p-value of a modified Fischer's exact test of the overrepresentation analysis; %List = percent of DAGs in the GO term considered by DAVID; %Genome = Percent of genes in the GO term from DAVID genome; Fold Enrichment =%List/%Genome; Fisher's test evaluate the question if genes in %List is more than random chance comparing to the genes in the %genome, i.e. H0: %List=%Genome, or Fold Enrichment = 1; Genes = EntrezID of the DAGs in the GO term.

S6 Table. **Brain-specific functional annotation clusters**.

Annotation Cluster 1	Enrichment Score: 2.09, average p-value=0.00813		
**Category**	**Term**	**Count**	**Gene ID**
GOTERM_BP_ALL	GO:0031175~neuron projection development	12	*CNTN4, DSCAML1, ITGA1, LIFR, MAP1B, NCAM2, NRXN3, PRKG1, RASGRF1, RELN, RXRA, SLIT3*
GOTERM_BP_ALL	GO:0048666~neuron development	14	*CNTN4, DSCAML1, FARP2, ITGA1, LIFR, MAP1B, NCAM2, NRXN3, OPCML, PRKG1, RASGRF1, RELN, RXRA, SLIT3*
GOTERM_BP_ALL	GO:0030182~neuron differentiation	16	*CNTN4, DSCAML1, FARP2, ITGA1, KCNMA1, LIFR, MAP1B, MDGA2, NCAM2, NRXN3, OPCML, PRKG1, RASGRF1, RELN, RXRA, SLIT3*
GOTERM_BP_ALL	GO:0007399~nervous system development	29	*ACSBG1, CHL1, CNTN4, DLL1, DSCAML1, FARP2, FGF11, FGF14, HNF1B, ITGA1, KCNMA1, LIFR, MAP1B, MDGA2, NAV1, NCAM2, NHLH2, NLGN2, NRXN3, OPCML, PAX5, PCSK2, PRKG1, RASGRF1, RELN, RXRA, SLIT3, TCF7L2, XRCC5*
GOTERM_BP_ALL	GO:0048699~generation of neurons	18	*CNTN4, DLL1, DSCAML1, FARP2, ITGA1, KCNMA1, LIFR, MAP1B, MDGA2, NCAM2, NRXN3, OPCML, PRKG1, RASGRF1, RELN, RXRA, SLIT3, XRCC5*
GOTERM_BP_ALL	GO:0048812~neuron projection morphogenesis	10	*CNTN4, DSCAML1, ITGA1, LIFR, MAP1B, NCAM2, NRXN3, RELN, RXRA, SLIT3*
GOTERM_CC_ALL	GO:0043005~neuron projection	13	*ATP1A2, CLCN2, CNTN4, ERC2, GRIN2B, ITGA1, KCNMA1, LRP1, MAP1B, NCAM2, RASGRF1, RELN, RXRA*
GOTERM_BP_ALL	GO:0022008~neurogenesis	18	*CNTN4, DLL1, DSCAML1, FARP2, ITGA1, KCNMA1, LIFR, MAP1B, MDGA2, NCAM2, NRXN3, OPCML, PRKG1, RASGRF1, RELN, RXRA, SLIT3, XRCC5*
GOTERM_BP_ALL	GO:0007409~axonogenesis	8	*CNTN4, DSCAML1, MAP1B, NCAM2, NRXN3, RELN, RXRA, SLIT3*
GOTERM_BP_ALL	GO:0048667~cell morphogenesis involved in neuron differentiation	8	*CNTN4, DSCAML1, MAP1B, NCAM2, NRXN3, RELN, RXRA, SLIT3*
GOTERM_BP_ALL	GO:0007411~axon guidance	4	*CNTN4, NRXN3, RELN, SLIT3*
GOTERM_CC_ALL	GO:0030424~axon	5	*CNTN4, KCNMA1, MAP1B, NCAM2, RXRA*
GOTERM_BP_ALL	GO:0007417~central nervous system development	9	*CNTN4, DSCAML1, HNF1B, MAP1B, MDGA2, NHLH2, PRKG1, RELN, SLIT3*
GOTERM_BP_ALL	GO:0007420~brain development	5	*CNTN4, DSCAML1, HNF1B, PRKG1, RELN*

Annotation Cluster 3	Enrichment Score: 1.69, average p-value=0.02042		
**Category**	**Term**	**Count**	**Gene ID**
GOTERM_CC_ALL	GO:0045202~synapse	14	*ATP1A2, CADPS, ERC2, GABRG3, GRID1, GRIN2B, HOMER2, ITPR1, KCNMA1, MAP1B, MUSK, NLGN2, PCDH15, SYNGR1*
GOTERM_CC_ALL	GO:0043005~neuron projection	13	*ATP1A2, CLCN2, CNTN4, ERC2, GRIN2B, ITGA1, KCNMA1, LRP1, MAP1B, NCAM2, RASGRF1, RELN, RXRA*
GOTERM_CC_ALL	GO:0044456~synapse part	10	*ERC2, GABRG3, GRID1, GRIN2B, HOMER2, ITPR1, KCNMA1, MAP1B, NLGN2, SYNGR1*
GOTERM_BP_ALL	GO:0048167~regulation of synaptic plasticity	5	*CNTN4, GRIN2B, MAP1B, RASGRF1, SYNGR1*
GOTERM_BP_ALL	GO:0050804~regulation of synaptic transmission	7	*CNTN4, GRIN2B, KCNMB4, MAP1B, NLGN2, RASGRF1, SYNGR1*
GOTERM_BP_ALL	GO:0051969~regulation of transmission of nerve impulse	7	*CNTN4, GRIN2B, KCNMB4, MAP1B, NLGN2, RASGRF1, SYNGR1*
GOTERM_BP_ALL	GO:0031644~regulation of neurological system process	7	*CNTN4, GRIN2B, KCNMB4, MAP1B, NLGN2, RASGRF1, SYNGR1*

The genes supporting brain-specific GO terms of the functional annotation clustering are listed.

S7 Table. **TopGO terms analysis**.

Ontology	GO ID	Term	Annotated	Significant	Expected	p-value
BP	GO:0007268	synaptic transmission	621	23	10.58	4.30E-05
	GO:0070588	calcium ion transmembrane transport	139	6	2.37	5.50E-05
	GO:0071872	cellular response to epinephrine stimulus	8	0	0.14	6.20E-05
	GO:0007417	central nervous system development	674	14	11.48	9.90E-05
	GO:0006898	receptor-mediated endocytosis	171	6	2.91	0.00012
	GO:0035249	synaptic transmission, glutamatergic	69	5	1.18	0.00013
	GO:0034765	regulation of ion transmembrane transport	268	7	4.56	0.00017
	GO:0048813	dendrite morphogenesis	72	1	1.23	0.00018
	GO:0048148	behavioral response to cocaine	11	1	0.19	0.00019
	GO:0002040	sprouting angiogenesis	48	0	0.82	0.00044
	GO:0006112	energy reserve metabolic process	147	4	2.5	0.00049
	GO:0016525	negative regulation of angiogenesis	53	0	0.9	0.00058
	GO:0001657	ureteric bud development	80	1	1.36	0.00063
	GO:0086091	regulation of heart rate by cardiac cond...	19	0	0.32	0.00067
	GO:0051966	regulation of synaptic transmission, glutamatergic	41	3	0.7	0.00089
	GO:0051968	positive regulation of synaptic transmission, glutamatergic	16	2	0.27	0.001
	GO:0090288	negative regulation of cellular response to growth factor stimulus	88	1	1.5	0.00108
	GO:2000311	regulation of alpha-amino-3-hydroxy-5-methyl-4-isoxazole propionate selective glutamate receptor activity	18	2	0.31	0.00109
	GO:0021756	striatum development	14	0	0.24	0.0011
	GO:0071805	potassium ion transmembrane transport	134	4	2.28	0.00111
	GO:0007205	protein kinase C-activating G-protein coupled receptor signaling pathway	32	4	0.55	0.00117
	GO:2000310	regulation of N-methyl-D-aspartate selective glutamate receptor activity	13	2	0.22	0.00144
	GO:0060402	calcium ion transport into cytosol	90	1	1.53	0.0016
	GO:0030282	bone mineralization	82	3	1.4	0.00185
	GO:0086064	cell communication by electrical coupling involved in cardiac conduction	17	1	0.29	0.00193
	GO:0006605	protein targeting	416	5	7.09	0.00201
	GO:0016477	cell migration	852	17	14.51	0.00209
	GO:1901381	positive regulation of potassium ion transmembrane transport	10	0	0.17	0.00211
	GO:0086013	membrane repolarization during cardiac muscle cell action potential	8	0	0.14	0.0023
	GO:0032092	positive regulation of protein binding	47	1	0.8	0.00235
	GO:0051480	cytosolic calcium ion homeostasis	192	3	3.27	0.00252
	GO:0060612	adipose tissue development	26	0	0.44	0.00276
	GO:0007411	axon guidance	319	13	5.43	0.00284
	GO:0050974	detection of mechanical stimulus involved in sensory perception	20	1	0.34	0.00306
	GO:0060291	long-term synaptic potentiation	27	1	0.46	0.00308
	GO:0052695	cellular glucuronidation	16	1	0.27	0.00313

GO:0005977	glycogen metabolic process	62	3	1.06	0.00313
GO:0050919	negative chemotaxis	17	1	0.29	0.00315
GO:0050796	regulation of insulin secretion	139	3	2.37	0.00319
GO:0030032	lamellipodium assembly	43	1	0.73	0.0033
GO:0007612	learning	97	4	1.65	0.0033
GO:0032321	positive regulation of Rho GTPase activity	120	6	2.04	0.00335
GO:0014068	positive regulation of phosphatidylinositol 3-kinase signaling	44	1	0.75	0.00341
GO:0010765	positive regulation of sodium ion transport	19	1	0.32	0.00367
GO:0021879	forebrain neuron differentiation	40	0	0.68	0.00369
GO:0007613	memory	77	5	1.31	0.00377
GO:0038084	vascular endothelial growth factor signaling pathway	19	0	0.32	0.00384
GO:0086014	atrial cardiac muscle cell action potential	8	0	0.14	0.00405
GO:0035235	ionotropic glutamate receptor signaling pathway	22	2	0.37	0.00431
GO:0060071	Wnt signaling pathway, planar cell polarity pathway	25	1	0.43	0.00436
GO:0007202	activation of phospholipase C activity	52	1	0.89	0.00483
GO:0001993	regulation of systemic arterial blood pressure by norepinephrine-epinephrine	8	0	0.14	0.00484
GO:0043647	inositol phosphate metabolic process	48	3	0.82	0.00486
GO:0030048	actin filament-based movement	89	4	1.52	0.00495
GO:0060055	angiogenesis involved in wound healing	12	0	0.2	0.00496
GO:0003007	heart morphogenesis	184	7	3.13	0.00496
GO:0043271	negative regulation of ion transport	61	0	1.04	0.00503
GO:0021854	hypothalamus development	17	0	0.29	0.00584
GO:0060337	type I interferon signaling pathway	64	4	1.09	0.0059
GO:0030101	natural killer cell activation	60	2	1.02	0.00612
GO:0046548	retinal rod cell development	8	0	0.14	0.0062
GO:0046470	phosphatidylcholine metabolic process	54	3	0.92	0.00621
GO:0000186	activation of MAPKK activity	54	0	0.92	0.00635
GO:0006883	cellular sodium ion homeostasis	11	1	0.19	0.00661
GO:0007512	adult heart development	15	0	0.26	0.00661
GO:0007626	locomotory behavior	164	8	2.79	0.00698
GO:0045454	cell redox homeostasis	44	1	0.75	0.00758
GO:0072215	regulation of metanephros development	18	1	0.31	0.0077
GO:0010822	positive regulation of mitochondrion organization	51	2	0.87	0.00779
GO:0007216	G-protein coupled glutamate receptor signaling pathway	13	2	0.22	0.00781
GO:0006814	sodium ion transport	154	3	2.62	0.00786
GO:0060122	inner ear receptor stereocilium organization	17	0	0.29	0.0079
GO:0001523	retinoid metabolic process	65	3	1.11	0.00818
GO:0048843	negative regulation of axon extension involved in axon guidance	8	1	0.14	0.00825
GO:0050732	negative regulation of peptidyl-tyrosine phosphorylation	27	0	0.46	0.0083
GO:2000649	regulation of sodium ion transmembrane transporter activity	23	0	0.39	0.00832
GO:0001568	blood vessel development	460	10	7.83	0.00863

	GO:0090316	positive regulation of intracellular protein transport	98	2	1.67	0.00874
	GO:0090330	regulation of platelet aggregation	11	1	0.19	0.00922
	GO:0060251	regulation of glial cell proliferation	12	0	0.2	0.00927
	GO:1902547	regulation of cellular response to vascular endothelial growth factor stimulus	8	0	0.14	0.00935
	GO:0030730	sequestering of triglyceride	12	1	0.2	0.00952
	GO:0050890	cognition	192	9	3.27	0.00972
	GO:0007158	neuron cell-cell adhesion	12	4	0.2	0.00975
	GO:0034205	beta-amyloid formation	9	0	0.15	0.00976
	GO:0035556	intracellular signal transduction	1902	40	32.39	0.00988
	GO:0055003	cardiac myofibril assembly	14	1	0.24	0.00998
CC	GO:0030054	cell junction	883	21	15.08	6.40E-06
	GO:0045211	postsynaptic membrane	158	7	2.7	7.10E-06
	GO:0005581	collagen trimer	76	3	1.3	7.20E-05
	GO:0042734	presynaptic membrane	50	3	0.85	0.00011
	GO:0016324	apical plasma membrane	210	3	3.59	0.00022
	GO:0014069	postsynaptic density	107	4	1.83	0.00026
	GO:0045202	synapse	459	16	7.84	0.00035
	GO:0030425	dendrite	291	12	4.97	0.00035
	GO:0008328	ionotropic glutamate receptor complex	42	2	0.72	0.00096
	GO:0048786	presynaptic active zone	22	1	0.38	0.00174
	GO:0030027	lamellipodium	127	2	2.17	0.00197
	GO:0005891	voltage-gated calcium channel complex	34	0	0.58	0.00253
	GO:0030666	endocytic vesicle membrane	97	2	1.66	0.00287
	GO:0043025	neuronal cell body	263	5	4.49	0.00339
	GO:0005887	integral component of plasma membrane	1075	25	18.36	0.0038
	GO:0030424	axon	245	3	4.18	0.00389
	GO:0032154	cleavage furrow	37	0	0.63	0.00411
	GO:0043235	receptor complex	242	6	4.13	0.00446
	GO:0005737	cytoplasm	7567	133	129.22	0.00525
	GO:0000159	protein phosphatase type 2A complex	18	0	0.31	0.00625
	GO:0008076	voltage-gated potassium channel complex	63	2	1.08	0.0065
	GO:0043197	dendritic spine	77	3	1.31	0.00687
	GO:0005884	actin filament	49	1	0.84	0.00711
	GO:0012507	ER to Golgi transport vesicle membrane	31	1	0.53	0.00711
	GO:0014701	junctional sarcoplasmic reticulum membra...	9	0	0.15	0.00728
	GO:0048471	perinuclear region of cytoplasm	447	6	7.63	0.00771
	GO:0042613	MHC class II protein complex	10	0	0.17	0.0082
	GO:0001725	stress fiber	43	0	0.73	0.00936
MF	GO:0005516	calmodulin binding	150	3	2.54	0.00023
	GO:0005001	transmembrane receptor protein tyrosine phosphatase activity	18	1	0.31	0.0003

GO:0017124	SH3 domain binding	96	1	1.63	0.00033
GO:0008013	beta-catenin binding	53	1	0.9	0.00048
GO:0044325	ion channel binding	70	0	1.19	0.00052
GO:0004970	ionotropic glutamate receptor activity	17	2	0.29	0.00073
GO:0016641	oxidoreductase activity, acting on the CH-NH2 group of donors, oxygen as acceptor	10	1	0.17	0.00076
GO:0030165	PDZ domain binding	87	1	1.48	0.00098
GO:0035254	glutamate receptor binding	22	2	0.37	0.00104
GO:0008331	high voltage-gated calcium channel activity	9	0	0.15	0.00125
GO:0008270	zinc ion binding	947	13	16.06	0.00165
GO:0015020	glucuronosyltransferase activity	26	1	0.44	0.00176
GO:0016500	protein-hormone receptor activity	8	0	0.14	0.00202
GO:0005102	receptor binding	1072	25	18.18	0.00223
GO:0004871	signal transducer activity	1306	27	22.15	0.00223
GO:0070016	armadillo repeat domain binding	9	1	0.15	0.00285
GO:0004714	transmembrane receptor protein tyrosine ...	62	2	1.05	0.00301
GO:0001965	G-protein alpha-subunit binding	14	1	0.24	0.00349
GO:0030506	ankyrin binding	20	0	0.34	0.00357
GO:0005234	extracellular-glutamate-gated ion channel	17	2	0.29	0.00412
GO:0004896	cytokine receptor activity	61	3	1.03	0.00437
GO:0005249	voltage-gated potassium channel activity	78	1	1.32	0.00469
GO:0005524	ATP binding	1202	25	20.38	0.00497
GO:0005044	scavenger receptor activity	34	1	0.58	0.00533
GO:0034185	apolipoprotein binding	14	1	0.24	0.00564
GO:0005089	Rho guanyl-nucleotide exchange factor ac...	62	4	1.05	0.00627
GO:0045295	gamma-catenin binding	12	1	0.2	0.00642
GO:0034483	heparan sulfate sulfotransferase activit...	13	1	0.22	0.00646
GO:0043548	phosphatidylinositol 3-kinase binding	17	0	0.29	0.00734
GO:0030159	receptor signaling complex scaffold acti...	19	1	0.32	0.00768
GO:0031701	angiotensin receptor binding	8	1	0.14	0.00846
GO:0005245	voltage-gated calcium channel activity	40	1	0.68	0.00934

Results using the Kolmogorov-Smirnov (K-S) test and "weight01" GO decorrelation method. The GO terms are ranked for "weigth01" p-values across GO categories (biological process [BP], cellular component [CC] and molecular function [MF]). Legend: Annotated = number of genes in the GO term; Significant = number of significant genes in the GO term; Expected = number of expected by random chance genes in the GO term; Pvalue= p-value in K-S test with "weigth01" GO decorrelation.

S8 Table. **Centres contributing FTD samples**.

Site PI	Main Institution
Barbara Borroni	Department of Medical Sciences, Neurological Clinic, University of Brescia, Italy
Valeria Novelli*	Department of Molecular Cardiology, IRCCS Fondazione S. Maugeri, Pavia, Italy
Giacomina Rossi	Neurological Institute Carlo Besta, Milan, Italy
Daniela Galimberti*	Department of Neurological Sciences, Dino Ferrari Institute, University of Milan, Italy
Innocenzo Rainero	Department of Neuroscience, University of Torino, Italy
Luisa Benussi	Molecular Markers Laboratory, IRCCS Istitute, Fatebenefratelli, Brescia, Italy
Benedetta Nacmias	Department of Neuroscience, Psychology, Drug Research and Child Health, University of Florence, Florence, Italy
Amalia Bruni	Regional Center of Neurogenetic, Lamezia Terme, Italy

* = PI representing multiple centers

Summary of the 8 research groups that contributes samples to this study.
